# A Phenomic Perspective on Factors Influencing Breast Cancer Treatment: Integrating Aging and Lifestyle in Blood and Tissue Biomarker Profiling

**DOI:** 10.3389/fimmu.2020.616188

**Published:** 2021-02-01

**Authors:** Ainhoa Arana Echarri, Mark Beresford, John P. Campbell, Robert H. Jones, Rachel Butler, Kenneth J. Gollob, Patricia C. Brum, Dylan Thompson, James E. Turner

**Affiliations:** ^1^ Department for Health, University of Bath, Bath, United Kingdom; ^2^ Department of Oncology and Haematology, Royal United Hospitals Bath NHS Trust, Bath, United Kingdom; ^3^ Department of Medical Oncology, Velindre Cancer Centre, Cardiff, United Kingdom; ^4^ Department of Cancer and Genetics, Cardiff University, Cardiff, United Kingdom; ^5^ South West Genomics Laboratory Hub, North Bristol NHS Trust, Bristol, United Kingdom; ^6^ International Center for Research, A.C.Camargo Cancer Center, São Paulo, Brazil; ^7^ School of Physical Education and Sport, University of São Paulo, São Paulo, Brazil

**Keywords:** breast cancer, tumors, clinical response, biomarkers, immunosenescence, lifestyle, exercise, physical activity

## Abstract

Breast cancer is the most common malignancy among women worldwide. Over the last four decades, diagnostic and therapeutic procedures have improved substantially, giving patients with localized disease a better chance of cure, and those with more advanced cancer, longer periods of disease control and survival. However, understanding and managing heterogeneity in the clinical response exhibited by patients remains a challenge. For some treatments, biomarkers are available to inform therapeutic options, assess pathological response and predict clinical outcomes. Nevertheless, some measurements are not employed universally and lack sensitivity and specificity, which might be influenced by tissue-specific alterations associated with aging and lifestyle. The first part of this article summarizes available and emerging biomarkers for clinical use, such as measurements that can be made in tumor biopsies or blood samples, including so-called liquid biopsies. The second part of this article outlines underappreciated factors that could influence the interpretation of these clinical measurements and affect treatment outcomes. For example, it has been shown that both adiposity and physical activity can modify the characteristics of tumors and surrounding tissues. In addition, evidence shows that inflammaging and immunosenescence interact with treatment and clinical outcomes and could be considered prognostic and predictive factors independently. In summary, changes to blood and tissues that reflect aging and patient characteristics, including lifestyle, are not commonly considered clinically or in research, either for practical reasons or because the supporting evidence base is developing. Thus, an aim of this article is to encourage an integrative phenomic approach in oncology research and clinical management.

## Introduction

Breast cancer is the most common form of cancer affecting women worldwide, with around two million new cases each year (1). Breast cancer is the second most common form of cancer overall and the fifth most common cause of cancer-specific death (2). Men diagnosed with breast cancer account for 1% of all malignancies and represent 1% of all cases of breast cancer worldwide (3). The risk of developing breast cancer is influenced by many factors, including age, age at first birth, parity, breast feeding, menopausal status, physical activity level, body composition, and hereditary factors (e.g., mutations in key genes, such as BRCA1) (4). Treatment for breast cancer has improved over the last four decades and can consist of a combination of traditional and more advanced interventions including surgery, chemotherapy, radiation therapy, hormone therapy, small molecule therapy, immunotherapy and other targeted approaches (such as mTOR inhibitors) (5). Although most treatments are very effective, the clinical profile and characteristics of each patient are unique and tumor heterogeneity—even among patients with the same TNM (T: tumor, N: node, M: metastasis) staging—results in patient-to-patient variation in clinical outcomes. This patient-to-patient variation in clinical outcomes might partly be due to genetic factors, including germline mutations (e.g., BRCA1/2 or P53) or polymorphisms in genes encoding drug metabolizing enzymes and transporters (e.g., DPYD, TPMT or UGT1A1, involved in 5-fluorouracil, mercaptopurine or irinotecan metabolism, respectively) (6–8). Although these factors can be assessed, a challenge that remains is predicting which patients will respond optimally to different treatment options, and to stratify patients to provide the best care (9). Difficulties in managing heterogeneity in the clinical response exhibited by patients emphasizes the need to consider other factors when measuring and interpreting predictive and prognostic biomarkers in breast cancer.

Biomarkers are molecular, histological, radiographical or physiological characteristics that can be measured as an indicator of normal biological processes, pathogenic processes, or responses to an exposure or intervention, including therapy (10). From a clinical perspective, a biomarker could be described as an objective observation of the medical state of a patient, which can be assessed accurately and reproducibly (11). To be reliable, biomarkers need to be sensitive and specific. Sensitivity refers to the ability of the biomarker to correctly identify patients with a disease from the whole population, and specificity refers to the ability of the biomarker to correctly identify people without the disease (12, 13). Molecules linked with the presence of cancer are often referred to as tumor biomarkers or tumor antigens, where antigens are molecules containing sites that are recognized by, and interact with, components of the immune system. Neoantigens are antigens that are generated by somatic mutations in the tumor, whereas tumor-associated antigens can also be found in healthy tissues, usually at lower levels (14). Many classical tumor biomarkers are proteins, and they can either be located on the cell surface, in the intracellular space or secreted into body fluids by cancer cells or other local cells in response to the tumor(s) (2, 15). Further, many tumor biomarkers are shared among different cancers with only a few biomarkers being disease specific (2).

Tumor biomarkers can be categorized based on their role and time of assessment, including diagnostic, monitoring, predictive or prognostic biomarkers (16, 17). Diagnostic biomarkers, for example, show utility in early phases of disease, as they confirm the presence of a tumor, whereas biomarkers used for monitoring disease become more relevant following diagnosis and during treatment, as their serial measurement gives real-time information of disease status (16). In this review, our focus is largely on predictive and prognostic biomarkers, given their utility in establishing the clinical response to treatment. Predictive biomarkers assess the response or lack of response to a specific form of therapy, while prognostic biomarkers can reflect the natural course of the disease and thus can assess clinical outcomes in the absence of therapy (18). When interpreting any type of biomarker, the specific endpoint of analysis should also be taken into consideration (19). Examples include calculations of progression-free survival or objective response rates. The increasing relevance of biomarkers in the management of cancer has led to the development of a number of agencies who support and advise on the clinical use of biomarkers, including the American Society of Clinical Oncology (ASCO) (20–24), the American National Academy of Clinical Biochemistry (NACB) (25), the European Group of tumor markers (EGTM) (5, 26), the European Society of Medical Oncology (ESMO) (27, 28), the National Institute for Health and Care Excellence (NICE) and the National Comprehensive Cancer Network (NCCN). Guidelines have also been produced with the validation steps needed for biomarkers to reach the clinics, including evaluation of confounding factors, analytical and clinical validation, demonstration of clinical utility and regulatory approval (29). Further, a biomarker registry has been created to compile data from ongoing, completed but not yet published, and completed studies, as well as those with negative results, serving as a useful tool for further analyses or for the design of new biomarker studies (30).

The search for new cancer biomarkers continues, and once measurements become established, there is often further validation and refinement, including the assessment of other biomarkers simultaneously, to improve the sensitivity or specificity of tests. An additional step, often not undertaken either for practical reasons or because the supporting evidence base is developing, is understanding whether cancer biomarkers are influenced by broader factors, including the characteristics of patients and their lifestyle. If it could be established, that factors such as age, physical activity level, or body composition, influence the concentration or characteristics of a given biomarker, then accounting for these inter-individual patient-centric factors, might improve the clinical utility of that measurement^1^. Given that first; some biomarkers indicate the severity of disease and are secreted or expressed by tumor cells during active disease (31–34), and that second; factors, such as exercise, physical activity or body composition, are known to influence disease progression (35–40), then it is conceivable that there is interaction. Indeed, the effects of age, exercise, and adiposity, on the composition and function of cells, tissues and organs, is well established, and there are a variety of mechanistic links with disease (41, 42). In turn, the composition and characteristics of tissues that are both local and distant to tumor sites, could influence the measurement of cancer biomarkers and also disease progression directly.

The first part of this article summarizes current and emerging breast cancer biomarkers that are measured in tumors or in blood (see **Tables 1A**, **1B** and **Table 2**). The second part of this article summarizes the effects that aging, exercise or physical activity, and adiposity can have, on the cellular composition and function of a variety of cells and tissues, including tumors. In places, links between these broader characteristics of patients and overall cancer risk, disease progression, and treatment outcomes are highlighted. In summary, the overall aim of this article is to encourage an integrative phenomic approach in oncology research and clinical management.

Table 1AMeasurements made in tumours: singleplex/duplex/quadruplex assays.

**Table:** 

Biomarker or test	Type	Detection technique	RTCs, meta-analyses and other studies	Reviews, and consensus papers	Recommendations	Used clinically
**ER**	Singleplex	Semiquantitative IHC, FFPE	(31, 43–44)	(2, 45, 46)	ASCO (20): treatment decisions EGTM (26): treatment decisions ESMO (27, 28): treatment decisions NACB (25): treatment decisions; prognosis (combined biomarkers)	Yes
**PR**	Singleplex	IHC, FFPE	(47–48)	(49, 50)	ASCO (20): treatment decisions EGTM (26): treatment decisions ESMO (27, 28): treatment decisions NACB (25): treatment decisions; prognosis (combined biomarkers)	Yes
**HER2**	Singleplex	IHC, FISH, sequencing; FFPE	(51, 52–53–55)	(56, 57)	ASCO (20): treatment decisions EGTM (26): treatment decisions ESMO (27, 28): treatment decisions NACB (25): treatment decisions; prognosis if combined	Yes
**UPA** and **PAI-1**	Duplex	ELISA, fresh/frozen tissue, FFPE	(32, 58–59)	(60, 61)	ASCO (20): prognosis newly diagnosed (node−) ASCO (62): treatment decisions (adjuvant therapy ER+HER2−node−) EGTM (26): prognosis (combined biomarkers); prediction (adjuvant therapy ER+HER2−node−) ESMO (27, 28): prognosis (node−/+); treatment decisions (combined biomarkers; early disease) NACB (25): prognosis; further evaluation for treatment decisions	*Note (A)*
**P53**	Singleplex	IHC, TTGE/sequencing, cDNA microarrays; FFPE	(63, 64–65)	(66, 67)	ASCO (20, 22): insufficient data for treatment decisions EGTM (5): insufficient data ESMO (27, 28): not mentioned NACB (25): prognosis (conflicting results) 2007 3^rd^ international workshop on TP53: prognosis	No
**Ki-67**	Singleplex	IHC, RT qPCR fresh/frozen tissue, FFPE	(68–69)	(70, 71)	ASCO (20): insufficient data for prognosis ASCO (62): not recommended for treatment decisions EGTM (26): prognosis if combined ESMO (28): prognosis ER+HER2−, treatment decisions (combined biomarkers; adjuvant therapy) NACB (25): not mentioned International Ki67 BC working group (72): guidelines on use	Yes
**D cathepsin**	Singleplex	IHC (FFPE); immunoenzymatic or radiometric assays (tumour lysates); Western Blotting	(73–74)	(75, 76)	ASCO (20): insufficient data for prognosis/prediction EGTM (5): insufficient data ESMO (27, 28): not mentioned NACB (25): prognostic (node−; conflicting results)	No
**PSA**	Singleplex	IHC (FFPE), ELISA (tumour cytosolic extracts)	117	(77–78)	ASCO (20, 22): not mentioned for BC EGTM (5, 26): Not mentioned for BC ESMO (27, 28): Not mentioned for BC NACB (25): not mentioned for BC	No
**IHC4**	Quadruplex (ER, PR, HER2 and KI67)	IHC, FFPE	(79, 80)		ASCO (22): Not recommended for treatment decisions EGTM (5, 26): not mentioned ESMO (27, 28): not mentioned NACB (25): not mentioned 2018 NICE DG34 guidelines: not recommended for treatment decisions in early ER+HER2-node- (uncertain analytical validity)

**Table 1B:** Measurements made in tumours: multiplex assays.

Biomarker or test	Type	Detection technique	RTCs, meta-analyses and other studies	Reviews, and consensus papers	Recommendations	Used clinically
**TILs**	Multiplex	Microscopy, ICC, flow cytometry, gene expression; blood, fresh/frozen tissue, FFPE	(81–82)		ASCO (22): insufficient evidence for treatment decisions EGTM (5, 26): not mentioned ESMO (28): prognosis, not treatment decisions NACB (25): not mentioned 2014 International TILs working group (83): guidelines/recommendations 2019 St Gallen Consensus: prognosis	No
**Oncotype DX**	Multiplex (21 genes)	RT PCR, FFPE	(84–85)		ASCO (20, 22): prognosis/prediction (adjuvant therapy, tamoxifen) EGTM (26): prognosis/prediction (adjuvant therapy, tamoxifen ER+HER2− ESMO (28): prognosis/prediction (adjuvant therapy; combined biomarkers) NACB (25): prognosis/prediction (adjuvant therapy; combined biomarkers) 2018 NICE DG34 guidelines: treatment decisions in early ER+HER2-node- (adjuvant chemotherapy)	Yes
**Mammaprint**	Multiplex (70 genes)	Microarray, fresh/frozen tissue, FFPE	(86–87)		ASCO (23): prognosis/treatment decisions EGTM (26): prognosis/treatment decisions (adjuvant therapy; invasive) ESMO (28): prognosis/prediction adjuvant therapy; combined biomarkers) NACB (25): not mentioned 2018 NICE DG34 guidelines: not recommended for treatment decisions in early ER+HER2-node- (not cost effective) FDA approved	Yes
**Prosigna**	Multiplex (50 genes)	Microarray, FFPE	(88–89)		ASCO (22): treatment decisions (adjuvant therapy, combined biomarkers, ER+HER2−node−) EGTM (26): prognosis/ treatment decisions (adjuvant therapy, combined biomarkers, ER+HER2−) ESMO (28): prognosis/prediction (adjuvant therapy, combined biomarkers) NACB (25): not mentioned 2018 NICE DG34 guidelines: treatment decisions in early ER+HER2-node- (adjuvant chemotherapy) FDA approved	Yes
**Endopredict**	Multiplex (8 genes)	RT PCR, FFPE	(90–91)		ASCO (22): treatment decisions (adjuvant therapy ER+HER2−node−) EGTM (26): prognosis/treatment decisions (adjuvant therapy, combined biomarkers, ER+HER2−) ESMO (28): prognosis/prediction (adjuvant therapy, combined biomarkers) NACB (25): not mentioned 2018 NICE DG34 guidelines: treatment decisions in early ER+HER2-node- (adjuvant chemotherapy) Not FDA approved but approved for use in Europe	Yes
**Rotterdam signature**	Multiplex (76 genes)	Microarray, fresh/frozen tissue	(92–93)		ASCO (20): insufficient data EGTM (26): insufficient data ESMO (28): not mentioned NACB (25): not mentioned Not commercially available	No

ASCO: American Association of Clinical Oncology; BC: Breast Cancer; Chemo: chemotherapy; EGTM: European Group of Tumour Markers; ELISA: Enzyme Linked Immunosorbent Assay; ER: Estrogen receptor; FDA: Food and Drug Administration; ESMO: European Society of Medical Oncology; FISH: Fluorescence In Situ Hybridization; FFPE: formalin-fixed paraffin-embedded tissue; HER2: Human Epidermal Growth Factor Receptor 2; ICC: Immunocytochemistry; IHC: Immunohistrochemistry; NACB: American National Academy of Clinical Biochemistry; NICE: National Institute for Health and Care Excellence; PR: Progesterone receptor; PSA: Prostate Specific Antigen; RT PCR: reverse transcription Polymerase Chain Reaction; RT qPCR: Quantitative reverse transcription Polymerase Chain Reaction; TILs: Tumour Infiltrating Lymphocytes; TTGE: temporal temperature gradient gel electrophoresis; UPA and PAI: Urokinase plasminogen activator and Plasminogen Activator Inhibitor 1. Notes: (A) Not widely used as fresh or freshly frozen tissue is required.

**Table 2:** Measurements made in blood: singleplex and multiplex assays.

Biomarker or test	Type	Detection technique	RTCs, meta-analyses and other studies	Reviews, and consensus papers	Recommendations	Used clinically
**CEA**	Singleplex	ELISA, plasma/serum	(94–95)	(2)	ASCO (20, 21): monitor treatment (combined biomarkers, metastatic) EGTM (5): prognosis (combined biomarkers, early recurrence) ESMO (27, 28): not mentioned NACB (25): monitor treatment (combined biomarkers)	Occasionally
**CA 15.3** **&** **CA 27.29**	Singleplex (one or the other)	ELISA, plasma/serum	(33, 96–97)		ASCO (20, 21): monitor treatment (combined biomarkers, metastatic) EGTM (5): prognosis (combined biomarkers, early recurrence) ESMO (27, 28): not mentioned NACB (25): monitor treatment (combined biomarkers)	Occasionally
**MCA**	Singleplex	ELISA, plasma/serum	(98, 99)		ASCO (20, 22): Not mentioned (favor: CA15.3 & CA 27.29) EGTM (5): Not mentioned (favor: CA15.3 & CA 27.29) ESMO (27, 28): not mentioned NACB (25): not mentioned	No
**Circulating HER2**	Singleplex	ELISA, plasma/serum	(100, 101–102)		ASCO (20, 22): insufficient evidence prognosis/treatment EGTM (5, 26): Not mentioned ESMO (27, 28): Not mentioned NACB (25): Potential: prognosis/treatment/ prediction/monitoring (undergoing evaluation) FDA approved	No
**Circulating PSA**	Singleplex	Immunoassays, serum	(103, 104)	(105–106, 107)	ASCO (20, 22): not mentioned for BC EGTM (5, 26): Not mentioned for BC ESMO (27, 28): Not mentioned for BC NACB (25): not mentioned for BC	No
**ctDNA**	Multiplex	PCR or sequencing techniques, blood	(108, 109)	(107)	ASCO/CAP (24): complementary to genomic tests (metastasis), insufficient evidence (early-stage/monitoring/recurrence) EGTM (5, 26): not mentioned ESMO (27, 28): not mentioned NACB (25): not mentioned FDA approved (PIK3CA mutation test) (110)	No
**CTCs**	Multiplex	Microscopy, flow cytometry, RT-PCR, blood	(34, 111–112)	(113)	ASCO (20, 22): insufficient evidence for treatment decisions EGTM (5, 26): not mentioned ESMO (27, 28): not mentioned NACB (25): prognosis/monitoring (advanced disease, undergoing evaluation) FDA approved (CellSearch assay) (114)	No
**Circulating Immune cells**	Multiplex	Flow cytometry, blood	(115–117)	(118, 119)	No	No

ASCO: American Association of Clinical Oncology; BC: Breast Cancer; CAP: College of American Pathologists; CEA: Carcinoembryonic Antigen; CTCs: Circulating Tumour Cells; ctDNA: Circulating tumour DNA; EGTM: European Group of Tumour Markers; ELISA: Enzyme Linked Immunosorbent Assay; FDA: Food and Drug Administration; ESMO: European Society of Medical Oncology; HER2: Human Epidermal Growth Factor Receptor 2; HE4: Human epididymis protein; MCA: Mucin-like carcinoma associated antigen; NACB: American National Academy of Clinical Biochemistry. PSA: Prostate Specific Antigen.

## Measurements in Tumors

### Estrogen Receptor (ER)

Estrogen receptors (ERs) are nuclear steroid receptors that operate as transcriptional regulators of several cell processes, such as proliferation and differentiation, in response, primarily, to estrogen (45). There are two forms, ER-alfa and ER-beta, and the majority of ER-positive tumors express the alfa form (49, 120). ER expression is measured by semiquantitative immunohistochemistry in formalin-fixed paraffin-embedded tumor biopsies (46). ER expression has proven importance as a prognostic and predictive factor by identifying which patients will respond to hormone therapies (e.g., aromatase inhibitors, tamoxifen and other ER antagonists) informing treatment decisions, and providing an estimate of overall survival (2). For almost 50 years, many studies have confirmed both the prognostic and the predictive value of ER measurements (31, 43) and ER status is used widely in clinics after diagnosis. For example, a study analyzed data from 4478 breast cancer patients across seventeen cancer registries in six European countries, to determine the influence of hormone receptor status on survival (121). Comparing ER status and relative survival over 5 years, it was found that women who had been classified as ER positive had better outcomes (90% survival, 95% CI: 88–92) compared to ER-negative counterparts (77% survival; 95% CI: 73–78). Among ER-positive women, tamoxifen treatment was associated with a 10% decrease in relative excess risk of death compared to women not treated with tamoxifen. Although the majority of studies examining ER have focused on the alfa form, some reports have shown prognostic value of the beta isoform, even in ER-alpha negative tumors (44, 122). Attention has been directed more recently to mutations in the gene that encodes the ER—so called ESR1 mutations—because they have been associated with resistance to endocrine therapy, especially in metastatic settings (62).

### Progesterone Receptor (PR)

Progesterone receptors (PRs) are also nuclear steroid receptors that govern processes such as proliferation and differentiation in response, primarily, to progesterone. There are two isoforms, PR-alpha and PR-beta, which regulate different genes (50). PR expression is measured by semiquantitative immunohistochemistry in formalin-fixed paraffin-embedded tumor biopsies. In healthy breast tissue, both isoforms are expressed equally, but some studies have shown a dysregulation of this balance in breast cancer (47). A large literature base supports the use of PR status for predicting clinical outcomes. For example, a study defined both clinical utility and cut off points of immunohistochemistry for PR status measurement in a ‘test’ group of 1235 cases of primary breast cancer receiving endocrine therapy. This study then confirmed clinical utility for successful therapeutic outcomes in an extra ‘validation’ group of 423 breast cancer patients who underwent mastectomy and were randomized to either 5 years of adjuvant tamoxifen treatment or no adjuvant treatment (123). Analysis of formalin-fixed samples from the 423 patients showed that PR was a strong and significant predictive factor of both improved disease-free and overall survival (HR = 0.546, P = 0.0034; HR = 0.595, P = 0.0040 respectively). The PR-alpha/PR-beta ratio has also been suggested to influence responsiveness to hormone therapies, with some studies showing that a high ratio of PR-alpha to PR-beta expression is linked to tamoxifen resistance (48). Combined with information from assessing ER-status, it is known that tumors expressing both ER and PR respond best to endocrine therapies (49).

### Human Epidermal Growth Factor Receptor 2 (HER2)

Human epidermal growth factor receptor 2 (HER2; also known as c-erbB-2, due to the encoding gene, or HER2/neu, due to its discovery in neuroblastoma rat models (51)), is an epithelial growth factor oncoprotein, localized in the cell membrane and involved in communication among cells for proliferation, differentiation and survival signalling (2). HER2 is commonly measured in formalin fixed sections of tumor tissue, by immunohistochemistry, but also by Fluorescence In Situ Hybridization (56). HER2 status is most commonly used to identify patients eligible for treatment with HER2-targetting therapies such as trastuzumab, also known as herceptin (22, 52). HER2 status has prognostic and predictive value, in part, due to the effectiveness of HER2-targetting therapies. However, HER2 positivity and overexpression has been associated with worse prognosis and reduced disease-free and overall survival in the absence of HER2-targetting treatments (124). In addition, HER2 expression has been associated with resistance to endocrine therapy, especially tamoxifen (53, 57, 125, 126) but has been linked with the success of other chemotherapy regimens. For example, a study including 638 patients with ER and/or PR negative tumors and axillary lymph node involvement, showed that patients with HER2 overexpression benefited from chemotherapeutic regimens where anthracycline-based drugs such as doxorubicin were added, compared to HER2 negative patients. The 10-year disease free survival of HER2 positive patients increased from 26% to 41% when treated with doxorubicin, whereas survival did not change in the HER2 negative group (40 vs. 41%) (54). In another study, 442 women with node positive breast cancer were randomized to three different doses of adjuvant chemotherapy, combining cyclophosphamide, doxorubicin and fluorouracil. Women with tumors overexpressing HER2 (≥50% overexpression) benefited the most from high doses of chemotherapy, compared to those with little or no expression of HER2 (55).

### Urokinase Plasminogen Activator (uPA) and Plasminogen Activator Inhibitor 1 (PAI-1)

Urokinase plasminogen activator (uPA) is a serine protease that converts plasminogen into plasmin, which has a key role in degradation of extracellular matrix-components, leading to release of growth factors implicated in migration and invasion (60, 61). The proteolytic activity of uPA is regulated by inhibitors such as plasminogen activator inhibitor 1 (PAI-1). Given the role of uPA in metastasis, PAI-1 was once thought to be protective, but studies have shown that this inhibitor is also associated with tumorigenesis, likely by preventing apoptosis (58) or enhancing angiogenesis (32). Simultaneous measurement of both molecules has been shown to have better prognostic and predictive value compared to measuring them separately (127). Both uPA and PAI-1 are commonly measured in parallel with enzyme-linked immunosorbent assays (ELISA) in extracts of the primary tumor, and general reference cut off levels are 3 ng/mg and 14 ng/mg respectively. uPA and PA-1 levels have prognostic value in breast cancer patients regardless of menopausal status (128) and node status (129, 130), and high levels of both markers have been significantly associated with shorter overall and disease-free survival. A prospective randomized control trial showed that uPA and PAI-1 levels also had predictive value, identifying lymph-node negative breast cancers with better responses to adjuvant chemotherapy consisting of cyclophospamide, methotrexate and 5-fluorouracil (CMF) (131). In this study, breast cancer patients were stratified into either a high-risk or low-risk group, depending on whether they had high or low levels of uPA and PAI-1, respectively. Among the high-risk group, patients receiving chemotherapy had a 44% decrease in the relative risk of disease recurrence compared to those who did not receive treatment (RR = 0.56, 95% CI: 0.25–1.28). Similar findings have been reported in other studies (59), and future studies need to confirm clinical utility with other more commonly used treatment regimens (132).

### Tumor Protein 53 (P53)

Tumor protein P53 is a nuclear protein involved in cell cycle regulation that also acts as a tumor suppressor, binding to DNA in the presence of damage and triggering either DNA repair pathways, checkpoint arrest or apoptosis (66). In tumors, one or both alleles of P53 are commonly deleted and/or mutated (63), and this can result in non-functional P53, which, unable to detect DNA damage, contributes to tumorigenesis. Overexpression of mutated versions of P53 can promote tumor formation due to oncogenic gain-of-function activity (67). Traditionally P53 status is examined by immunohistochemistry in formalin fixed paraffin blocks, which is useful for identifying overexpression. However, given the importance of identifying specific mutations, Temporal Temperature Gradient Gel Electrophoresis, with sequencing of aberrant migrating bands to determine the nature of mutations, or cDNA microarrays are now more common. Overexpression of P53 protein and some mutations have been linked with poor prognosis and shorter survival (64, 133–137). For example, there was a significant reduction in disease free survival over 5 years among 700 women with node-negative breast cancer exhibiting tumors that were positive for a mutated version of P53. Disease free survival probability at 5 years was 80% for P53 negative tumors, 72% for P53 positive tumors with low expression, and 58% for P53 positive tumors with high expression (P < 0.05) (133). Some studies have supported the predictive value of P53 for treatment outcomes, as certain mutations (e.g., stop codons, point or deletion mutations, in regions like the zinc-binding domain) have been associated with resistance to some forms of chemotherapy (e.g., doxorubicin, tamoxifen, 5-fluorouracil and mitomycin, or cyclophosphamide, methotrexate and 5-florouracil) or radiotherapy (138–144). Other studies on the other hand, have shown better responses to certain chemotherapy regimens (e.g. paclitaxel, or epirubicin and cyclophosphamide) among patients with mutations in P53, such as deletions, transversions or transitions in exons 4, 6, 8 or 10 (65, 145).

### Ki-67

Ki-67 is a nucleic protein that is a marker of proliferation expressed at higher levels during mitosis (70). It is commonly assessed by immunohistochemistry, typically using the MIB-1 antibody (71), although examining gene expression using RT qPCR provides comparable results (68). High Ki-67 expression in tumor tissue is associated with poorer outcomes (146–149). For example, a metanalysis of 12,155 breast cancer patients showed that, in the overall population, Ki-67 expression was associated with decreased overall (HR 1.95, 95% CI: 1.70–2.24; P < 0.001) and disease-free survival (HR 1.93, 95% CI: 1.74–2.14; P < 0.001) (146). Similar results have been shown by other studies, examining patients undergoing endocrine therapy (150). On the other hand, some studies have shown that positive responses to certain forms of therapy can be predicted with high Ki-67 scores, such as some chemotherapy combinations (e.g., docetaxel, fluorouracil and epirubicin) in ER positive tumors (151) or addition of adjuvant chemotherapy to endocrine therapy in HER2 negative tumors (152). However, other studies have not been able to prove predictive value of Ki-67 (69, 153). The International Ki-67 in Breast cancer working group reviewed the available evidence base and provided guidelines for the accurate measurement of this marker (72).

### D Cathepsin

D cathepsin is a lysosomal aspartyl protease that breaks down intracellular and endocytosed proteins in most mammalian cells (75) and is involved in remodeling processes in mammary tissue (76). D cathepsin can be assessed by immunohistochemistry in formalin fixed paraffin embedded tumor samples, or immunoenzymatic assays and radiometric immunoassays in breast tumor lysates or by Western Blotting. Some studies have indicated that D cathepsin has prognostic value in primary breast cancer. For example, in an analysis of 2810 cytosolic extracts of breast tissue by radiometric immunoassay, it was shown that tumors with high levels of D cathepsin had significantly poorer relapse-free and overall survival regardless of node or menopausal status (73). In addition, dividing D cathepsin levels into four quartiles (Q1: 0–33, Q2: > 33–47, Q3: > 47–70, and Q4: > 70 pmol/mg protein) an association was shown between patients in the higher quartiles with early relapse and death. Relapse-free survival probability at 10 years in the group with highest D cathepsin levels was 36% compared to 55% among the group with the lowest levels. In addition, overall survival probability was 43% in the group with the highest levels compared to the 63% in the group with the lowest levels. Although other studies have shown similar results (154–156), the prognostic value of D cathepsin has not been fully established and is not used routinely. However, some studies have shown associations with treatment outcomes, as patients with higher levels seem to benefit from tamoxifen-based therapies (157, 158) but other studies show no impact (74, 159).

### Prostate-Specific Antigen (PSA)

Prostate-specific antigen (PSA) is a serine protease with chymotrypsin-like activity which is normally released from the prostate into seminal fluid to increase sperm motility. PSA is most commonly considered to be a serum biomarker for the diagnosis, prognosis and progression of prostate adenocarcinomas. However, PSA is also produced by other tissues, including the breast, and PSA has received attention in breast cancer (103). PSA can be detected by different methods, such as immunoassays in tumor cytosolic extracts, or immunohistochemistry and studies have shown prognostic value in breast cancer (77, 160). For example, a study of 174 breast cancer patients measured PSA in samples of tumor cytosol and found that PSA positive tumors correlated with early disease stage, smaller tumors and estrogen receptor positivity (77). Moreover, patients with PSA-positive tumors showed a significantly lower risk of relapse and death. However, other studies have not been able to confirm independent prognostic value for PSA (161, 162). Studies have also linked PSA to treatment outcomes. For example, in an analysis of tumor cytosol from 434 patients with breast cancer that had recurred who were treated with tamoxifen, a significant association was shown between high PSA and poor treatment response, as well as poor progression-free and overall survival (P < 0.001) (78). Further research is needed to confirm the clinical utility of PSA in breast cancer.

### IHC4

Immuno-HistoChemical-4 score (IHC4) is a four-parameter immunohistochemistry test that measures the ER, PR, HER2 and Ki-67 in formalin fixed paraffin embedded tumor samples. In 2011, the ATAC trial (Arimidex, Tamoxifen, Alone or in Combination) examined the prognostic value of combining those four immunohistochemistry markers among 1125 ER positive breast cancer patients in comparison with another multiparameter test—Oncotype DX, or Genomic Health Recurrence Score—covered in the next section (79). A prognostic model and a combined score, the IHC4 score was computed. Results showed independent prognostic value of each of the immunohistochemical markers, and a prognostic value for the IHC4 score that was comparable to that of Oncotype DX (although IHC4 score was slightly more prognostic for distant recurrences). In turn, the IHC4 score was subsequently examined and validated in an additional group of 786 ER positive patients. High levels of the adjusted IHC4 score were shown to be a strong prognostic factor for negative outcome (HR = 4.1, 95% CI: 2.5–6.8). Other later studies have confirmed the utility of IHC4 to identify ER positive breast cancer patients that have a low risk of recurrence (80). However, the IHC4 test needs further validation and investigation in large randomized trials before it can be used routinely in clinical practice.

### Tumor Infiltrating Lymphocytes (TILs)

Tumor infiltrating lymphocytes (TILs) reflect the immune response to the presence of a tumor (81). Most studies have focused on the predictive value of T cells, but many other immune cell subtypes are present within tumors, including natural killer cells, B cells and, despite the common name referring to “lymphocytes”, macrophages have also received attention (163). TILs can be detected by several methods including immunocytochemistry, flow cytometry, gene expression and semiquantitative histological evaluation by light microscopy (164). The frequency of TILs varies among the different breast cancer subtypes, and TILs are typically most abundant in the most aggressive forms, such as basal-like (ER−PR−HER−) and HER2-positive tumors (165). Studies have shown that infiltration of some lymphocyte sub-types, such as cytotoxic CD8+ T cells and helper CD4+ T cells, B cells and dendritic cells, are associated with good prognosis and therefore longer survival. However, studies have also shown that the infiltration of other cells, including regulatory T cells, neutrophils, and tumor-associated macrophages (TAMs) with an M2-like (alternatively activated) phenotype are associated with worse prognosis (82, 164, 166–168). Studies have examined the predictive value of TILs in the context of treatment outcomes, showing significant associations between high frequencies of TILs and positive responses to anthracycline-based chemotherapy (166), or to chemotherapy combined with trastuzumab (81). TILs have received a lot of attention in research settings, and studies have interpreted results in a variety of ways, including examining the presence or absence of cell subtypes, and also their relative abundance. The international TILs working group meeting in 2013 produced guidelines for these assessments, yet further work is required for routine clinical use (83).

### Oncotype DX

Oncotype DX, developed by Genomic Health (California, USA; now part of Exact Sciences, Wisconsin, USA), is a multiparameter RT-PCR assay that simultaneously measures the expression of 21 genes in formalin-fixed paraffin embedded tumor samples. The panel of genes includes 16 cancer-related genes, such as HER2 and ER, and others implicated in proliferation and invasion, and also 5 genes for reference (84). Based on the relative expression of each gene, a recurrence score is computed classifying patients into three risk categories: low (recurrence score lower than 18), intermediate (recurrence score between 18 and 30), and high (recurrence score above 30). The assay was designed to predict risk in lymph node negative, ER positive breast cancer patients treated with tamoxifen. The prognostic value of the Oncotype DX recurrence score has been extensively validated. High scores are associated with shorter relapse-free and overall survival in both lymph node positive and lymph node negative patients (84, 85, 169–172). The predictive value of this test is best demonstrated by identifying patients with ER positive tumors who would benefit most from adjuvant chemotherapy, regardless of node involvement (170, 172). A study tested the 21-gene recurrence score assay in tumor samples from the phase III trial SWOG-8814, which included lymph node negative ER-positive breast cancer patients treated with either tamoxifen alone or with chemotherapy consisting of cyclophosphamide, doxorubicin and fluorouracil prior to tamoxifen (172). The study confirmed the significant prognostic value of the assay in the tamoxifen alone group as shown by previous studies. The study also showed a significant improvement in survival from the addition of chemotherapy to tamoxifen in the high-risk score group, but a lack of benefit from chemotherapy in low-intermediate score groups. In the high risk group, the 10-year estimates for percentage of disease-free survival were 55% for chemotherapy and tamoxifen vs. 43% for tamoxifen alone (P = 0.033), and for overall survival, 68% for chemotherapy and tamoxifen vs. 51% for tamoxifen alone, (P = 0.027), and for breast-cancer specific survival, 73% for chemotherapy and tamoxifen vs. 54% for tamoxifen alone (P = 0.033). On this basis, Oncotype DX and similar platforms are routinely used to help decision-making for the use of adjuvant chemotherapy in ER-positive breast cancer. Further research is needed to evaluate the use of Oncotype DX among ER negative patients.

### MammaPrint

Mammaprint, developed by Agendia (Amsterdam, Netherlands), is a multi-parameter microarray-based technique that simultaneously measures the expression of 70 genes in either fresh or frozen tumor tissue or formalin fixed paraffin embedded samples, which produces a recurrence score. In turn, patients are classified as either low risk with a good prognosis signature, or high risk with a bad prognosis signature. Several studies have confirmed the clinical utility of this test to identify patients with better or worse outcome (86, 173, 174), and to inform clinical decisions over whether to treat patients with adjuvant chemotherapy following surgery (175, 176). One of these is the MINDACT study (Microarray in Node- Negative Disease may Avoid ChemoTherapy), a prospective randomized trial, which was conducted with 6693 patients diagnosed with early breast cancer. In this study, the recurrence risk calculated by Mammaprint and referred to as genomic risk was compared with risk calculated by an online decision-making tool for clinicians that was available at the time (Adjuvant!Online; referred to as clinical risk) (87, 176). Patients were assigned as being low or high risk with both scores. There were 1550 patients with high clinical risk (determined by the online tool) and low genomic risk (determined by MammaPrint) (176). After randomization to receive adjuvant chemotherapy or not following surgery, the difference in survival was small: 1.5% lower among patients not receiving chemotherapy. Indeed, the 5-year survival rate without distant metastasis was 95.9% (95% CI: 94.0–97.2) among those receiving chemotherapy compared to 94.4% (95% CI: 92.3–95.9) among patients who were not treated with chemotherapy. Thus, MammaPrint is a useful tool for informing treatment decisions.

### Prosigna

Prosigna (also called PAM50 gene signature) is a 50-gene microarray-based technique developed by Nanostring technologies (Seattle, USA) for use with fresh and frozen tissue or formalin fixed paraffin embedded samples. The test classifies tumors in one of four subtypes: Luminal A, Luminal B, HER2-positive or Basal-like (88). The test provides a Risk of Recurrence score (ROR), where low scores (ROR<40) categorize patients as being low risk, and high scores (ROR>60) categorize patients as being high risk. Several studies have validated the prognostic value of Prosigna in postmenopausal women with ER-positive early breast cancer. For example, a study of 1478 women from the ABSCSG-8 trial who were being treated with tamoxifen or tamoxifen and anastrozole, showed that the ROR score from Prosigna has significant prognostic value (177). This study showed that the Luminal A subtype presented a lower ROR score after 10 years compared with Luminal B, emphasizing the utility of this multiparameter test for predicting the risk of distant recurrence. Other studies have analyzed the utility of Prosigna for therapeutic prediction. For example, a randomized controlled study—the DBCG89D trial—among patients with early breast cancer treated with either CMF (cyclophosphamide, methotrexate and fluorouracil) or CEF (cyclophosphamide, epirubicin and fluorouracil) undertook Prosigna assays on samples from 686 patients and studied associations with distant recurrence, time to recurrence and overall survival (89). The results showed that patients from the HER2 subtype presented a significant benefit from anthracycline-based (epirubicin) chemotherapy, in comparison with patients from the luminal subtypes, as the time to distant recurrence was significantly longer in the HER2 subtype treated with CEF. Further, the benefit of CEF therapy over CMF was associated with higher ROR scores.

### Endopredict

Endopredict is an 8-gene RT polymerase chain reaction developed by Sividon Diagnostics (Koln, Germany, now part of Myriad Genetics). The test is usually used with formalin fixed paraffin embedded samples, and, combined with tumor size and nodal status, it can predict the clinical risk of distant recurrence ten years after diagnosis by assigning a score (either low or high). Endopredict is normally used as a prognostic test for patients with early breast cancer, who are ER-positive and HER2-negative (90, 178) however other studies have demonstrated its utility to inform treatment decisions. A retrospective comparative analysis of five large clinical trials (GEICAM/9906, GEI-CAM 2003/02, ABCSG-6, ABCSG-8 and TransATAC trials) analyzed a total of 3746 women, who were treated with either adjuvant endocrine therapy alone or endocrine therapy plus chemotherapy, and determined the ability of Endopredict to estimate the 10-year distant recurrence free interval rates (91). The results showed that women who received chemotherapy in addition to endocrine therapy and those who had higher Endopredict scores, showed significantly lower distant recurrence after 10 years compared to those who only received endocrine therapy.

### Rotterdam Signature

The Rotterdam signature is a multi-parameter microarray-based technique that analyses tumor expression, in fresh or frozen tissue, of 76 genes involved in cell death, cell cycle, proliferation, immune response, survival, cell to cell signaling, DNA replication and repair. These genes do not overlap with Oncotype DX or Mamma-Print. The Rotterdam signature—so called due to its development at the Erasmus Medical Centre in Rotterdam—was designed for lymph node negative breast cancer patients, to predict metastatic disease over a period of five years. The model was validated in 171 breast cancer patients and showed a significant difference of 40% between good and poor prognosis groups for distant-metastasis-free survival at 60 months and a difference of 27% between groups for overall survival. This test could predict distant tumor recurrence regardless of age, menopausal status and tumor size, and could identify patients with a better prognosis who could avoid adjuvant systemic therapy (92). Later studies validated the Rotterdam signature in a large cohort of node negative breast cancer patients, including those from the TRANSBIG trial (network of TRANSlational research by the Breast International Group) (93, 179). Further research is needed for this index to be used regularly in routine practice.

### Summary of Genetic Profiling Tests

Despite the value of the genetic profiling platforms described above (i.e. Oncotype DX, MammaPrint, Prosigna, Endopredict and Rotterdam Signature) to inform treatment decisions, these tests fail to predict recurrence in a fraction of patients, particularly in those with luminal subtypes (180). Thus, new or improved tools are needed to accurately predict recurrence and avoid undertreatment and overtreatment.

## Measurements in Blood

### Carcinoembrionic Antigen (CEA)

Carcinoembrionic antigen (CEA) is a cell surface glycoprotein which is a 641 amino acid polypeptide chain that can be released into blood by tumor cells. It is the most widely used tumor biomarker in clinical settings and for several cancers, particularly carcinomas of the bowel (2). This biomarker, normally assessed by Enzyme-linked Immunosorbent Assay (ELISA) in plasma or serum, has also received a lot of attention in breast cancer, as studies examining its prognostic value have shown that high levels are associated with poorer outcomes (94, 96, 181). For example, in a prospective study that measured pre-operative CEA levels in serum among 2062 breast cancer patients, it was shown that high levels of CEA (>5 µg/L) in 12.7% of the patients correlated with nodal involvement and larger tumors (96). In addition, an elevated CEA level was present in 56.3% of patients exhibiting cancer recurrence. Furthermore, CEA was found to be an independent prognostic factor for both disease free and overall survival regardless of node status. In addition, high CEA was associated with a high probability of metastasis, as all patients with >7.5 µg/L had recurrences during the follow up time. Other studies have examined the predictive value of CEA and high levels have been associated with poorer responses to therapy in patients with advanced disease. For example, in a study of 232 breast cancer patients with recurrent tumors following mastectomy, an increase of >2 ng/ml after the second cycle of the therapy correlated with shorter progression-free survival compared with those with lower/stable levels: 6.7 vs. 17.7 months, respectively (P < 0.001) (95). Furthermore, high CEA was associated with bone metastases. Despite these promising results, further studies are required before CEA is used widely in clinical practice.

### CA 15.3 and CA 27.29

CA 15.3 and CA27.29 are mucin-like glycoproteins that belong to the MUC1 family. Mucins (MUCs) are heavily glycosylated, high molecular weight glycoproteins with an aberrant expression profile in various malignancies. The names 15-3 and 27.29 refer to the specific monoclonal antibodies used for detection. CA 15.3 is most commonly used although CA 27.29 has been shown to have comparable utility (182). These biomarkers are normally measured by ELISAs, but also other commercially available kits, based on radio-, enzyme- or chemi-luminescence. Studies examining CA 15.3 have shown that high levels of this protein are associated with worse outcomes and shorter survival (97, 100, 183). For example, one study in 2004 recruiting 600 newly diagnosed breast cancer patients showed that increased levels of CA 15.3 prior to surgery (>30 units/L) were associated with shorter overall survival [hazard ratio (HR) = 2.16, CI, 1.55–3.03, P < 0.0001], regardless of the type of adjuvant treatment administered (183). Another study prospectively measured pre-operative serum levels of CA 15.3 in 2062 breast cancer patients (96). It was shown that high levels of CA 15.3 (>30 kU/L) in 19.6% of the patients correlated with nodal involvement and larger tumors. In addition, CA 15.3 was a significant prognostic factor for disease free survival in the absence of CEA. Furthermore, rising CA 15.3 assessed with serial blood samples also predicts poor outcomes. Studies have also confirmed the predictive value of these biomarkers with several types of cancer treatment, including chemotherapy. A retrospective study examined CA 15.3 for predicting response to treatment in 73 patients with locally advanced breast cancer and found that elevated levels prior to administering of primary chemotherapy were significantly associated with poor clinical and pathological response (33). Furthermore, if the elevated levels of CA 15.3 were sustained following treatment, this appeared to be an independent predictor of recurrence (P = 0.007). Another study with 232 breast cancer patients who had recurrent tumors following mastectomy, analyzed the associations between CEA and CA 15.3 and the response to therapy (95). This study found that increased levels of CA 15.3 (an increase of >15 U/ml) after the second cycle of therapy correlated with shorter progression-free survival compared with normal levels: 7.7 vs. 17.3 months, respectively for CA 15.3 (P < 0.0001). Furthermore, elevated levels also correlated with metastases in the bones. Current evidence does not justify the use of CA 15.3 and CA27.29 for monitoring responses to therapy.

### Mucin-Like Carcinoma Associated Antigen (MCA)

Mucin-like carcinoma associated antigen (MCA) is another measurement of MUC-1. Some studies have measured MCA with other circulating markers, such as CA 15.3, CEA and Tissue Polypeptide (TPA) (98). Testing two different cut off values for MCA (11 U/ml and 15 U/ml), it has been shown that MCA is more sensitive than CA 15.3, CEA or TPA (68 vs. 32%, 10%, 26% for cut off 11 U/ml and 53 vs. 32%, 16%, 42% for cut off 15 U/ml) but, less specific than CEA and CA15.3 (42% for cut off 11U/ml or 73% for cut off 15 U/ml vs. 96% and 97% respectively). Changes to MCA levels have been related to tumor response to therapy in metastatic patients and elevated pre-surgical levels seem to be associated with lower disease-free survival. For example, a study recruiting 548 participants consisting of 148 primary breast cancer patients, 150 with metastatic breast cancer, 50 patients with benign disease, and 200 participants with no clinically evident disease, showed an association between higher pre-surgical levels of MCA with lower disease-free survival, which appeared to be most significant in those with no nodal invasion. Also, in the metastatic breast cancer subgroup, decreases in MCA levels positively correlated with therapeutic response in 82% of the patients (99). However, few studies have evaluated the prognostic and predictive value of MCA individually, precluding its use clinically.

### Circulating HER2

The extracellular domain of human epidermal growth factor receptor 2, also known as extracellular circular domain or ECD, can undergo proteolytic cleavage and can be released into blood, and is commonly measured by ELISA. High levels of circulating HER2 levels have been associated with worse outcomes and poorer survival, therefore measurement of this protein is a useful prognostic marker (100, 101). For example, it has been shown that higher levels of circulating HER2 were associated with a 50% reduction in overall survival in metastatic breast cancer patients compared to lower levels (10.1 months, 95% CI: 5.2–13.6 vs. 20.2 months 95% CI: 15.0–28.6, P < 0.001) (100). Some studies have also shown utility of this marker to monitor cancer recurrence (184), and the predictive value has been shown by studies showing that patients with high circulating ECD levels, which were sustained through treatment, benefited less from trastuzumab. For example, in a study of 175 breast cancer patients from the GeparQuattro trial, a >20% decrease in circulating HER2 throughout the course of treatment was associated with a 60% chance of pathologic complete response compared to patients where this decrease was not achieved through therapy (185). Almost identical results have also been shown with the response to lapatinib treatment (102).

### Circulating PSA

After being secreted by breast cancer cells, PSA likely accumulates in the tumor microenvironment and eventually reaches peripheral blood. PSA has been measured in serum from breast cancer patients, primarily using immunoassays, and some studies have shown prognostic utility of this biomarker in breast cancer management (103, 105, 186). However, other studies have not been able to demonstrate prognostic value, despite promising results when PSA is measured in tissue samples from the tumor (187, 188). In general, using circulating PSA as a biomarker for breast cancer among women remains a challenge, as PSA levels are very low compared to men, and often undetectable (106). Although more sensitive assays are being developed (104), further research with large cohorts of patients is required before this marker is used routinely in breast cancer management.

### Circulating Cell-Free DNA (ctDNA)

Apoptotic and necrotic cells can secrete fragments of DNA into blood, referred to as cell free DNA or cfDNA. If it can be confirmed that this DNA has come from cancer cells, then this measurement is better known as circulating tumor DNA (ctDNA). ctDNA is present at a very low concentration in plasma and enables non-invasive serial assessments of tumor characteristics including, assessing point mutations and DNA methylation in key genes (107). ctDNA is assessed *via* next generation sequencing or PCR-based assays. Recently, a ctDNA assay measuring 110 alpha catalytic subunit of phosphoinositide 3-kinase (PIK3CA) mutations in HER2-negative breast cancer patients has recently obtained FDA approval (110). Indeed, studies have confirmed the utility of ctDNA to monitor metastatic disease. For example, a prospective study examined plasma from 30 breast cancer patients to compare ctDNA levels, circulating tumor cells and CA 15.3 levels (108). Using digital PCR and targeted deep sequencing, somatic mutations or structural variants in PIK3CA and TP53 genes were screened for, identified and quantified at different timepoints. It was shown that the concentration of PIK3CA and TP53 mutations in plasma significantly positively correlated with increases in tumor burden, with high levels reflecting progressive disease in 89% of the cases and being associated with shorter overall survival (P < 0.001). Furthermore, it was suggested that ctDNA analysis could be predictive of therapeutic response earlier than CA 15.3 and circulating tumor cells. Other studies have shown that measurement of ctDNA can identify mutations linked to resistance to certain treatments, such as anti-HER2 therapy, and therefore predict treatment failure (109). However, further research is needed *via* more high-quality prospective studies, and standardized methodology, before it is used routinely in all clinics.

### Circulating Tumor Cells (CTCs)

Circulating tumor cells (CTCs) can be found at very low frequency in blood and just a few CTCs per 10 ml of blood can predict an aggressive primary tumor or metastasis (20). CTCs are a heterogeneous group of cell types, such as epithelial tumor cells, epithelial-to-mesenchymal cells and cancer stem cells (29). Given their low frequency, enrichment procedures and highly sensitive assays are required to measure them, and CTCs can be quantified *via* microscopy, flow cytometry or using RT-PCR (113). One of the widely used techniques is the CellSearch Assay, which has had FDA approval for prognostic and predictive use in metastatic breast cancer (114). CellSearch identifies circulating epithelial tumor cells, defining the CTC phenotype as EpCAM+ (Epithelial cell adhesion molecule), Cytokeratins (8+, 18+, and/or 19+), DAPI+ and CD45−, and only counts intact cells (intact cell >4 microns). Other methods are used in research settings, including flow cytometry, RT-PCR, gene expression arrays, and Fluorescence In Situ Hybridization. A study recruiting 99 metastatic breast cancer patients, enumerated CTCs using CellSearch after the second cycle of chemotherapy and showed that patients with ≥5 CTCs per 7.5 ml of blood exhibited reduced overall survival (8.7 months vs. 38.5 months, P < 0.001) and reduced progression-free survival (3 months vs. 9.4 months, P = 0.001) compared with patients who had < 5 cells per 7.5 ml of blood (34). In addition, the clinical benefit rate was also considerably lower (44 vs. 77%, P = 0.0051). Similar results were obtained in another prospective study, with metastatic patients before they started a new line of treatment (111). Finally, some studies have shown that CTCs can predict early relapse after neoadjuvant chemotherapy and shorter overall survival (189) and can predict treatment outcomes (112). Further validation studies and standardization is required for integration in clinics.

### Immune Profiles

The phenotype and function of immune cells, as well as the T cell repertoire and diversity in blood, have been examined for predictive and prognostic utility in the context of breast cancer. While an individual’s immune profile prior to a cancer diagnosis might influence clinical outcomes, cancer itself and/or treatment of the disease might exacerbate immunosenescence, changing immune profiles, leading to poor outcomes (190). In a study of 88 breast cancer patients with metastasis treated with cyclophosphamide or paclitaxel based chemotherapy regimens, extensive immunophenotyping was conducted in peripheral blood using flow cytometry (115). It was shown that among patients treated with paclitaxel, higher frequencies of naïve CD4+ or CD8+ T cells (CD45RA+CD95−CD27+CD28+) were associated with worse prognosis, as they correlated with shorter breast cancer specific survival (CD8+: 28.7 vs. 12.6 months, HR = 0.32 95% CI: 0.15–0.67, P = 0.0028; CD4+: 29.4 vs. 15.1 months, HR = 0.45 95% CI: 0.22–0.91, P = 0.027). In these patients, however, higher frequencies of CD11c+ dendritic cells were linked to better outcomes (13.4 vs. 25.3 months, HR = 4.60 95% CI: 1.23–17.1, P = 0.023). In the cyclophosphamide-treated group, CD14+ monocytes were also associated with good prognosis. Another study of 89 women with metastatic breast cancer showed that a CD8+CD28− cells were significantly increased compared to age-matched healthy women, and the frequency of these cells negatively correlated with progression free survival. The median survival was on average 2 months less (P < 0.001) among patients with high frequencies of CD8+CD28− cells (≥24.0% of the CD8+ T cell pool) compared to patients with a lower frequency (<24.0%) (191).

Some studies have examined whether the capacity of T cells to recognize tumor-associated antigens is a predictive or prognostic factor in breast cancer, and in turn, whether other aspects of immunosenescence influence this response. For example, the frequency of regulatory T cells and Myeloid derived suppressor cells (MDSCs: Lin−CD14+HLA-DR−) and HER2-specific T cells were examined among 40 patients with breast cancer prior to treatment (192). Patients exhibiting HER2-reactive T cells with a lower frequency of MDSCs had a 100% rate of survival after 5 years, compared to 38% of patients without HER2-reactive T cells with higher frequencies of MDSCs (P = 0.03). Furthermore, patients without HER2-reactive T cells and with higher levels of regulatory T cells had a 50% chance of survival compared to 100% survival of patients who mounted an anti-HER2 response with lower frequencies of regulatory T cells (P = 0.03). This survival advantage appeared to be independent of metastases (192). Moreover, T cell receptor diversity and clonality was studied in a group of 26 breast cancer patients. It was shown that HER2-positive patients displayed greater highly expanded clone ratios among the CD8+ T cell repertoire and that greater heterogeneity during chemotherapy was associated with a better clinical response (116).

Finally, there is concern that the overall immune profile of individuals, especially those exhibiting signs of immunosenescence, could influence the effectiveness of some immunotherapies (193) such as the monoclonal antibodies atezolizumab and avelumab for treating breast cancer by targeting PD-L1 (Programmed death ligand 1). This ligand can be expressed by tumors and other local cells (e.g., fibroblasts, endothelial cells, antigen presenting cells, myeloid derived suppressor cells) and inhibit tumor infiltrating T cells and NK cells which express PD1. Perhaps counter-intuitively, although PD-L1 is generally expressed at low levels (around 10%) on tumor cells, it has been shown that expression level positively correlates with a higher pathological complete response rate to neoadjuvant chemotherapy (117). However, PD-L1 expression appears not to be a good predictor of the response to PD-L1 targeting therapies (118). Taken together, these findings emphasize the importance of a strong anti-tumor immune response, hence the development of anti-PD1 therapies which target T cells and NK cells directly, such as pembrolizumab (118). Indeed, the capacity to mount a strong anti-tumor response is likely to be influenced by the characteristics of the *patient* such as immunosenescence but also the characteristics of the *tumor* given that tumor mutational burden is a strong predictor of the effectiveness of anti-PD1/PD-L1 treatment (118, 119).

## The Relevance of Aging and Lifestyle for Cancer Biomarker Profiling and Disease Progression

### Aging Influences Tissues and Blood

Aging is a temporal and progressive decline in the integrity of different physiological systems in an organism, consisting of tissue-specific changes characterised by processes such as inflammation and cellular senescence (41). These changes affect the functional properties of most cells, tissues and organs. One feature of aging is a gradual accumulation and redistribution of adipose tissue and a change to its cellular composition (194). The accumulation of adipose tissue is prominent within the abdominal cavity, but ectopic deposition also occurs around organs and within skeletal muscle (195, 196). Aging contributes to dysfunction of adipose tissue, characterised by changes to the tissue microenvironment at structural and cellular levels, resulting in abnormal secretions derived predominantly, from adipocytes and resident immune cells (197). Changes to the tissue include adipocyte hypertrophy, hypoperfusion, hypoxia, impaired insulin signaling, and accumulation of macrophages with a pro-inflammatory phenotype and infiltration of other inflammatory immune cells, such as sub-populations of T cells. In turn, adipose tissue dysfunction contributes toward a change in physiology at a local level (e.g., effects on the surrounding tissues, which could include, tumors for example) but also at a systemic level (e.g., low-grade inflammation and insulin insensitivity). Aging is also associated with a decline in muscle mass, muscle strength and changes to the myokinome (198–200). This muscle secretome consists of many cytokines and other soluble mediators produced by skeletal muscle in response to contractions during exercise. These so-called “exercise factors” are released into the circulation and exert endocrine or paracrine functions in other cells, tissues or organs, which has relevance for disease risk and progression (201). Interleukin-6 (IL-6) is the most well-characterized myokine and its roles when secreted from muscle are considered to be positive rather than pro-inflammatory, and include promoting glucose uptake, insulin sensitivity, lipolysis and fatty acid oxidation. However, in other contexts IL-6 is considered a mediator of inflammation, and so this cytokine is sometimes referred to as being pleiotropic; whereby depending on the context and the site of production, it can be pro- or anti-inflammatory (202, 203).

Inflammation is a self-limiting process which consists of a complex network of chemical signals triggered in the presence of damage for healing purposes, upon infiltration of pathogens as part of an immune response, or due to adipose tissue dysfunction (204). Inflammation can directly affect pathogens, such as by C-Reactive Protein activating complement (205), interferons limiting viral replication or by stimulating other immune processes, including attracting immune cells (206). The term inflammaging refers to the sustained low-grade inflammation that is characteristic of aging, and consists of higher levels of cytokines, such as IL-6 and TNF-alpha, increased levels of glucocorticoids and decreased levels of insulin-like growth factor 1 (207). Inflammaging has also been associated with deregulation of the complement pathway and increased activation of coagulation processes (208). Inflammaging leads to, or is part of, the age-associated decline and functional deterioration of immune competency, referred to as immunosenescence (209). The most accepted hallmarks of immunosenescence are lower numbers of naïve T cells and higher numbers of memory T cells, particularly within the CD8+ T cell pool (210). Sustained antigenic stimulation due to viral infection, especially Cytomegalovirus (CMV), drives these changes among T cells, but some cells accumulate with age *per se* (211), or as a result of other infections or perhaps even sub-clinical malignant transformation (212–214). Further, aging leads to impaired function of neutrophils, dendritic cells and natural killer cells, and increased frequencies of regulatory T cells and myeloid-derived suppressor cells (215). Most of these changes are very evident and well established in blood, but research characterizing inflammatory and immunological processes in tissues is limited.

Although it is likely that key mechanistic links between aging, cancer risk and tumor progression feature within inflammatory and immunological processes, it is important to emphasise that aging affects the structure and function of almost all aspects of physiology (41). In principle, a positive development in cancer care would be to incorporate measurements of aging into routine clinical tests and decision making to provide an estimate of a patient’s biological age. Despite the quest for a single and easily measured biomarker of aging, a range of blood and tissue biomarkers would need to be assessed. Aside from inflammatory and immunological parameters, assessing age-associated changes to a variety of body systems might be recommended, including the cardiovascular system (e.g., blood pressure, homocysteine), metabolic health (e.g., cholesterol, glucose, leptin), the central nervous system (e.g., amyloid β42, Tau), the hypothalamic pituitary axis and sympathetic nervous system (e.g., cortisol, DHEA, IGF-1, adrenaline, noradrenaline) (216). In addition, a number of genetic markers have been proposed, such as particular alleles of apolipoprotein E, polymorphisms in the gene encoding angiotensin-converting enzyme, mutations in mitochondrial DNA, telomere length, and many epigenetic changes (216–218). Recent emphasis has been placed on measuring the accumulation of senescent cells with aging. For example, by assessing DNA damage pathways and cyclin-dependent kinase inhibitors (e.g., p16^INK4a^), characterizing a senescence-associated secretory phenotype and apoptosis resistance, or determining morphological changes, such as lysosome accumulation (e.g *via* beta-galactosidase activity) or plasma membrane disturbances (e.g., caveolin-1 upregulation) (219). Finally, it might be recommended that a panel of aging biomarker measurements are interpreted alongside integrated whole-body measurements of physical functioning and frailty (e.g., sit-to-stand tests, walking tests, muscle function tests) (220).

### Aging Influences Tumor Progression and Cancer Outcomes

Given the constellation of changes that happen over the life course as time elapses, both chronological and biological aging are associated with increased cancer risk. Older people are more likely to get cancer, the majority of cases occur in people over 65 years of age (221). Given that life expectancy has significantly increased in the last century (222), around 30% to 40% of patients with breast cancer are over 70 years of age (223), and yet this population is underrepresented in clinical trials (224). Older age is associated with faster disease progression, and more complications, including treatment resistance (225). Indeed, menopausal status has a very strong influence on breast cancer risk, tumor characteristics, and disease progression (226). Although poor outcomes among older adults might be influenced by late/delayed diagnosis and undertreatment, a variety of other age-associated mechanisms likely contribute, of which some, interact with inflammation.

Deregulation of normal inflammatory processes is characteristic of aging, including a sustained release of pro-inflammatory cytokines, which can damage cells, and lead to an accumulation of damaged cells in tissues, which could conceivably progress into a malignancy (215, 227). Moreover, reactive oxygen species released by neutrophils in inflammatory settings can also damage cells, by oxidizing proteins, lipids and DNA (228). Once a tumor has developed, the levels of some cytokines have been associated with worse outcomes among patients. This is the case of IL-6, for example, as high serum levels appear to be linked with higher rates of metastasis and shorter survival in breast cancer patients (229, 230). Indeed, mechanistic studies have implicated IL-6 treatment resistance. For example, an *in vitro* study of drug-sensitive and drug-insensitive breast cancer cell lines showed that IL-6 was present at a high concentration in the media of drug-insensitive cells, but absent in the media of drug-sensitive cells (231). In addition, pre-treatment of drug-sensitive cells with IL-6 for 10 days caused an 8–10 fold increase in the resistance to the chemotherapeutic agent doxorubicin, and when drug-sensitive cells were transfected to constitutively express the IL-6 gene, drug resistance was shown to be 70-fold higher as compared with the drug-sensitive cells. Thus, it is conceivable that inflammaging could be one explanation for the treatment resistance that is sometimes seen among older people.

While several cytokines have well-established pro-tumor effects (e.g., IL-1, IL-4, IL-6) and can be produced by tumors directly in an autocrine manner (232), not all cytokines contribute toward pro-tumor processes. Indeed, many cytokines may elicit anti-tumor effects, including IL-2, IL-12, IL-15, IL-21, IFN-alfa and Granulocyte-Macrophage Colony-Stimulating Factor GM-CSF  (233). Some of these cytokines have anti-inflammatory roles and can interfere with cancer progression, either by enhancing anti-tumor immunity—stimulating certain immune cells—or by exerting direct anti-proliferative or pro-apoptotic actions on tumor cells directly (234). These properties have been explored in cytokine-based immunotherapy trials, either as monotherapy or in combination with other therapeutic agents (235). IL-2, for example, promotes survival, expansion and differentiation of activated NK and T cells, and its use in immunotherapy is approved for the treatment of metastatic disease in renal cell carcinoma and melanoma (236). IFN-alfa has been shown to exert anti-proliferative, pro-apoptotic and anti-tumor activity on cancer cells, and is approved to treat Hairy cell leukemia, AIDS-related Kaposi’s Sarcoma, Chronic Myelogenous Leukemia, Malignant Melanoma and Follicular lymphoma (237). However, challenges remain with these therapies, including short half-life of the cytokines, low response rates and frequent adverse events with high doses (238). However, it is conceivable that in older adults who might exhibit lower basal levels of IL-2 or IFN-alfa, or might have an impaired capacity to produce these cytokines (239, 240), these individuals might exhibit a greater risk of cancer and poorer anti-tumor responses. Indeed, the shift to a pro-inflammatory phenotype is well-known with aging (241) and some evidence shows this profile is reversed in extremely old populations, termed ‘anti-inflammaging’ (242, 243).

More broadly, other aspects of an aging immune system have been linked with unexpected hospitalisations during chemotherapy and limited effectiveness of some treatments—in particular immunotherapies—among older people (244–246). It is thought these effects might be partly attributed to the reduction of the naïve T cell pool, as this translates into an impaired ability to recognise and eliminate malignant cells. In addition, the senescence associated secretory phenotype (SASP) that some cells in aging tissues adopt, characterized by aberrant production of a range of cytokines, growth factors, proteases, and chemokines, could also play a role in tumorigenesis and progression (247). Finally, studies have shown that other markers of immunosenescence, including high frequencies of CD8+CD28− T cells, regulatory T cells, and myeloid-derived suppressor cells are associated with shorter survival (191).

### Aging Influences Cancer Biomarker Profiles

Evidence shows that the levels and characteristics of some cancer biomarkers, that are routinely measured in tissues and in blood, can be influenced by aging, which could affect the interpretation of clinical measurements and treatment outcomes. For instance, cross-sectional studies have shown that simple biomarkers measured in plasma, which are implicated in cancer risk and disease progression, can be influenced by aging (and also other factors that change with aging, including physical activity and body composition). For example, 77 cancer and inflammatory biomarkers were assessed in plasma from 1005 individuals from the Northern Sweden Population Health Study, and the influence of 158 inter-individual factors, was assessed (248). The results showed that 18 factors including age had a significant influence on the levels of one or more of 52 of the 77 biomarkers (248). In another study, plasma IGF-1 and serum IGFBP-3 were assessed in samples from 364 women with intraepithelial neoplasia or early invasive breast cancer and compared to 376 unaffected women (249). Women with early breast cancer had 21% higher IGF-1 and 19% higher IGFBP-3 than unaffected women, however IGF-1 levels were negatively associated with age (and also BMI) across all groups (249). Similar relationships have been shown with other biomarkers, for example, preoperative serum levels of CEA were shown to significantly positively correlate with age at diagnosis and menopausal status (250).

Some of the strongest evidence of aging influencing cancer biomarkers comes from studies that have considered the menopause. For example, differences in tumor characteristics were examined among 428 pre- and post-menopausal women (251). Compared with post-menopausal women, pre-menopausal women had significantly larger tumors (21% of pre-menopausal women had tumors of >5cm of diameter vs. 12% of post-menopausal women, P = 0.047). In addition, pre-menopausal women were more likely to have lymph node metastasis (77% of pre-menopausal women had positive axillary lymph nodes vs. 56% of post-menopausal women, P < 0.001) and more likely to have a positive expression of estrogen and progesterone receptors (ER: 56% of pre-menopausal women had positive expression vs. 44% of post-menopausal women, P = 0.002. PR: 52 vs. 41%, respectively, P = 0.014). Finally, pre-menopausal women had tumors with a greater proliferative capacity as shown by the higher likelihood of KI-67 positivity (33% of pre-menopausal women were KI-67 positive vs. 22.8%, of post-menopausal women, P = 0.017). Post-menopausal women, on the contrary, had significantly higher likelihood of expression of HER2 (pre-menopausal women: 2% vs. post-menopausal women: 19%, P = 0.038). Menopausal status also influences treatment decisions, and post-menopausal women were significantly more likely to have breast conserving surgery (P = 0.004), chemotherapy (P = 0.007), radiotherapy (P = 0.008), and endocrine therapy (P = 0.025) than pre-menopausal women. These results highlight important differences in breast tumors depending on menopausal status, which translate into differences in treatment and outcomes. However, other studies have suggested that age itself may be a stronger determinant of biological and etiological heterogeneity in breast tumors than menopausal status (252).

Aging in general is associated with particular molecular subtypes of breast cancer and a differential expression of some tumor biomarkers. For example, a study evaluated several makers by immunohistochemistry in different subtypes of invasive breast cancer among two groups (162 women ≤40 years and 100 women ≥50 years) (253). The results showed that Triple Negative Breast Cancer and HER2 subtypes were more common among young women. Furthermore, young women were more likely to have ER-negative tumors overall (253). In this work, tumor size and characteristics (ER, PR, HER2, Ki-67 and p53) were also compared (253). tumors from younger women were found to be significantly larger than those from older women; approximately 1.03 cm larger on average (P = 0.01). In addition, there was a significant quantitative differential expression of the tumor biomarkers on the basis of age. Younger women presented with lower expression levels of ER and PR (25% lower for ER, P < 0.01 and 10% lower for PR, P = 0.03), and higher levels of Ki-67 and P53 overexpression (10% higher for Ki-67, P = 0.01 and 13% higher for P53, P < 0.01) compared with women in the older group. Another study evaluated the influence of both age and menopausal status on several prognostic biomarkers in 1226 patients with operable primary breast cancer (254). Patients were divided into four groups: ≤40 years, premenopausal >40 years, postmenopausal <75 years and ≥75 years. The results showed that youngest patients had a worse prognosis, which improved with increasing age. Younger patients had the highest infiltration of TILs (P < 0.001), greatest p53 and Ki-67 expression (both P = 0.01) and the lowest expression levels of ER (P < 0.001). Finally, ER was also influenced by menopausal status, as expression level was higher in postmenopausal women compared to pre-menopausal counterparts (P < 0.001). Similar results have been found in larger studies (255). For example, by assaying 3800 tumor samples, significant inverse correlations with age and biomarkers of tumor growth and genetic instability (e.g., Ki-67 and p53 positivity) and growth factor receptor over expression (e.g., ErbB2+ or EGFR+) were shown (all P = 0.05), and among ER+ tumors, ER expression was significantly positively correlated with age (P < 0.0001). Likewise, a potential age-related association between HER2 and PR was evaluated in a study that examined 1104 ER positive tumors (divided into two age groups, 173 women of ≤45 years and 931 women of >45 years). There was an inverse relationship between HER2 and PR only in the group of women >45 years old (P =0.001) (256).

There is an increasing interest on how factors such as age can affect TILs. A study examined TILs in young (35–45 years), middle-aged (55–65 years) and older (>70 years) patients with luminal B (ER+PR+HER2−) breast cancer (257). TILs were phenotyped using CD3, CD4, CD5, CD8, CD20, CD68 and FOXP3 with immunohistochemistry. The results showed that increasing age was associated with a decrease in the overall percentage of stromal TILs in biopsies (P = 0.025). In addition, age had a significant effect on the composition the tumor/immune infiltrate, including a lower density of certain immune cells identified using CD3, CD5, CD8 and CD20, which was significant in all tumor regions (P < 0.042). The proportions of CD8+ TILs also decreased significantly with age in all tumor regions (P < 0.0001). However, the distribution patterns of TILs across each tumor region did not differ with age. Likewise, another study quantified the abundance of the immune cell infiltrate (B cells, CD4+ and CD8+ T cells, neutrophils, dendritic cells and macrophages) in tumors using transcriptome datasets. It was shown that there were no significant differences in the frequency or composition of TILs between age groups (young group: <40 years, old group: ≥40 years), but high levels of TILs, and in particular, CD8+ T cells, were associated with better clinical outcomes (P < 0.04) in women under 40 years of age (258).

Other studies have examined whether the multi-parameter molecular profiling tests, including IHC4, Oncotype Recurrence Score (RS) and Prosigna Risk of Recurrence Score, are influenced by age (259). Data from 940 women in the transATAC trial was split across three age groups (group 1: ≤59.8 years, group 2: 59.8–68.2 years and group 3: >68.2 years). The results showed that the prognostic performance of all molecular scores significantly differed with age, with the lowest scores among older patients. For example, for both IHC4 and Oncotype RS, their prognostic value appeared to be strongest in the lowest age group or group 1 (IHC4: group 1 HR = 3.01, 95% CI: 1.99–4.53, vs. group 2: HR = 1.67, 95% CI: 1.23–2.26 vs. group 3: HR = 1.64, 95% CI: 1.25–2.15. Oncotype RS: group 1: HR = 2.16, 95% CI: 1.62–2.87 vs. group 2: HR = 1.39, 95% CI: 1.16–1.66 vs. group 3: HR = 1.38, 95% CI: 1.11–1.73). However, Prosigna had the most prognostic value in women between 60 and 68 years or group 2 (group 1: HR = 3.87, 95% CI: 2.21–6.78 vs. group 2:HR = 4.51, 95% CI: 2.87–7.10 vs. group 3: HR = 1.83, 95% CI: 1.28–2.60). The influence of age on other more recent biomarkers, including CTCs and ctDNA has also been examined. For example, one study has reported a significant positive association between older age and ctDNA positivity among 31 primary breast cancer patients scheduled for neoadjuvant chemotherapy (260).

### An Active Lifestyle Is Associated With Better Cancer Outcomes

In addition to the robust evidence linking a physically active lifestyle with a reduction in breast cancer risk (261), studies are beginning to show that both exercise and physical activity are beneficial during cancer treatment and in the years after. The terms “exercise” and “physical activity” are sometimes used interchangeably, and there is an important distinction that has implications for the recommendations made in a cancer setting. For example, the term ‘‘physical activity’’ includes leisure-time, occupational, home-based and transport-related activities, some of which, might be undertaken as normal activities of daily living. The term “exercise” refers to a component of physical activity (within the leisure-time domain) and comprises physical activities that are planned, structured, repetitive and undertaken for the purpose of improving or maintaining components of physical fitness and/or sporting performance (262). In many studies, individuals are referred to as being ‘‘active’’ or ‘‘inactive’’ and these terms infer that individuals undertake (or fail to undertake) a defined level of physical activity (e.g., such as the recommendations published by the World Health Organization). Overall, patients with cancer are advised to lead a lifestyle that is as active as symptoms allow, whether this is through structured exercise or being physically active *via* activities of daily living, and specific guidelines have been developed for all stages of disease (263–265). For example, in general, patients are recommended to undertake around 150 min of moderate-intensity physical activity each week, which if achieved in a structured way, could be in bouts of around 30 min on 5 days of the week. Alternatively, recommendations also promote around 75 min of vigorous physical activity per week and advise supplementing this aerobic exercise with strength training on at least two days of a week. These recommendations are largely based upon those advocated by the World Health Organization and other bodies for the general population (266). However, very recently, more specific recommendations have been developed for patients with cancer, focusing in particular, on structured exercise training (267). For example, unique recommendations have been made for patients with complications (e.g., metastases) and for targeting particular side-effects and symptoms of disease and treatment (e.g., anxiety, fatigue, lymphedema, physical function) (267). For example, to counter fatigue, aerobic exercise training at moderate intensity for at least 12 weeks, exercising for 30 min three times a week has been recommended. Whereas for other complications, such as lymphedema, supervised resistance exercise training in a progressive manner two or three times per week is recommended.

Aside from the distinction between structured exercise and physical activity, many studies have shown that leading a physically active lifestyle generally brings about benefits, but studies that have employed structured and supervised exercise training provide the strongest evidence. Benefits include limiting treatment toxicity and alleviating cancer-related symptoms such as fatigue, anxiety, depression, and improving quality of life (QoL), mood and self-esteem (268, 269). For example, a randomized and controlled trial investigated the effects of exercise training on QoL and cardiorespiratory fitness among 53 postmenopausal breast cancer survivors (270). Women were either assigned to an inactive control group (n = 28) or were asked to exercise on cycle ergometers three times per week for 15 weeks (n = 25). Exercise was shown to increase overall QoL by 9.1 points compared to 0.3 points from the control group (mean difference, 8.8 points; 95% CI: 3.6–14.0; P = 0.001). Further, exercise also increased peak oxygen consumption by 0.24 L/min, whereas this decreased by 0.05 L/min in the control group (mean difference, 0.29 L/min; 95% CI: 0.18–0.40; P < 0.001). Moreover, a meta-analysis investigated effects of exercise interventions on QoL, social functioning, and physical functioning of breast cancer survivors in 18 trials (exercise group = 602 participants; control group = 603 participants) (271). The pooled effect confirmed that exercise significantly improved QoL (SMD = 0.35; I2 = 61%; 95% CI: 0.15–0.54; P = 0.0004), social functioning (SMD = 0.20; I2 = 16%; 95% CI: 0.08 to 0.32; P = 0.001), and physical functioning (SMD = 0.32; I2 = 32%; 95% CI: 0.20–0.44; P < 0.00001). Remaining active during cancer treatment has also been shown to improve clinical outcomes (268) and to enhance the efficacy of various cancer treatments (272). Other studies have shown that high levels of physical activity are associated with improved survival and lower levels of cancer recurrence (35–37). The mechanisms underlying these observations have not been proven, however likely explanations include exercise and physical activity influencing the effectiveness of treatment and modulating the properties of tumors both indirectly and directly.

### An Active Lifestyle Might Lead to Better Cancer Outcomes Due to Improved Chemotherapy Completion Rates

Patients who remain active during the period when they receive chemotherapy are more likely to tolerate a greater dose and complete their treatment (273, 274). For example, a study evaluated the potential benefits of aerobic and resistance exercise among 243 breast cancer patients undergoing adjuvant chemotherapy (273). Patients were randomly assigned to either supervised resistance exercise (n = 82), supervised aerobic exercise (n = 78) or usual care (n = 82), for a median of 17 weeks. Chemotherapy completion rate was assessed as the average relative dose intensity (RDI) from the originally planned regimen, and it is known that patients who receive an RDI of >85% have better outcomes. It was shown that patients in the resistance exercise training and the aerobic exercise training groups had better completion rates when compared to the usual care group, although this was only statistically significant for the resistance exercise regimen (RDI =84.1% control group vs. RDI= 89.8% resistance exercise group; mean difference=5.7%; 95% CI: 0.4–11.0; P < 0.033). Another study with a comparable group of breast cancer patients (n = 230) also compared usual care with two exercise regimens: a low intensity home based regimen and a moderate-high intensity supervised regimen combining aerobic and resistance exercises during a period of chemotherapy treatment (274). This study evaluated chemotherapy and trastuzumab completion rates and found that moderate-high intensity exercise improved completion rates, as a significantly lower number of patients in this group required chemotherapy dose adjustments compared to other groups (12% moderate-high intensity vs. 34% low-intensity vs. 34% usual care, P < 0.002). In addition, a smaller percentage of patients in the moderate-high intensity group required a delay or termination of trastuzumab therapy compared to the other two groups (6% moderate-high intensity vs. 24% low-intensity vs. 28% usual care). It is worth highlighting that the home-based exercise was not supervised and was of lower intensity, whereas the most effective intervention employed exercise that was supervised and of moderate intensity. Generally, supervised exercise, and activities that are more demanding, elicit more robust effects.

### Exercise and Physical Activity Influence Cancer Biomarker Profiles

There is a need for further research examining whether exercise and physical activity influence cancer biomarker profiles. Most evidence in support of this concept shows that broader factors, which are not necessarily cancer-specific, but are linked to clinical outcomes, including immune competency, inflammation, and metabolic health, can change among patients who modify their lifestyle (190). For example, a systematic review of 45 articles, including a variety of observational studies and randomized control trials of different designs, summarized the effects that physical activity in general can have among cancer survivors on biomarkers (275). This analysis included the HEAL (Health, Eating, Activity and Lifestyle) study, an observational prospective cohort study of 746 breast cancer survivors. It was concluded that regular physical activity can lead to immunological benefits (e.g., natural killer cell cytotoxicity, increased T cell proliferation), positive changes to proteins involved in insulin-signaling pathways (e.g., C peptide, insulin-like growth factors) and decreases in systemic inflammation (e.g., C-Reactive Protein, serum Amyloid A). Similar conclusions were drawn by a pooled analysis of three randomized controlled trials examining the influence of resistance exercise on factors that have been linked to poor cancer prognosis, including C-reactive protein, IL-6, IL1-beta, insulin-like growth factor binding proteins, leptin, serum amyloid A, adiponectin and TNF-alpha (276). Post-menopausal breast cancer survivors were allocated to either 1 year of resistance exercise consisting of two 1 hour supervised classes and one 45-minute home-based session each week (n = 109) or to a control group who undertook stretching and relaxation exercises (n = 106). It was shown by each trial that resistance training reduced systemic inflammation and improved insulin signaling.

A limited number of studies have examined the effects of exercise and physical activity on cancer-specific biomarkers. For example, a study of 15 females with breast cancer investigated the influence of 8 weeks of aerobic exercise training on serum levels of CEA and CA 15.3 (277). Participants exercised three times a week, at a light-to-moderate intensity. The results showed that participants exhibited a significant reduction in their BMI, body fat percentage, and body mass (P = 0.0001) and there was a trend for a decline in the levels of CA 15.3 (P = 0.091). There was no significant change in CEA. Another study, examined whether 12 weeks of structured exercise affected CEA among 54 healthy elderly women (70–77 years), randomized to different groups, varying on the frequency of exercise undertaken (278). The results showed that CEA significantly decreased in all groups with the largest decrease (percentage change: −59 ± 5%) among women who exercised 2–3 days per week.

### Exercise and Physical Activity Affect Tumors Directly and Indirectly

Exercise and physical activity lead to changes in tumor characteristics, including angiogenesis and enhanced tumor blood perfusion (due to an increase in tumor blood vessel density, function and maturity, which leads to reductions in intratumoral hypoxia), impaired growth and increased immune cell infiltration (279–283). These changes are clinically relevant as they may enhance the efficacy of some therapies, such as chemotherapy or immunotherapy, by facilitating the delivery of drugs to the tumor, and increased tumor vascularization and blood perfusion could facilitate immune-surveillance and processes such as reactive oxygen species production by some immune cells and treatments (272).

For example, a study in 50 athymic female mice evaluated the effects of 6 weeks voluntary wheel running on breast cancer growth and progression (279). Half of the mice were allocated to an active group with access to a running wheel and the other half were a control group with no access to a running wheel. Mice were implanted with human breast cancer cells on the first day of the study. During the intervention, tumor growth was monitored, as well as several markers of tumor blood perfusion, hypoxia, vascularization and angiogenesis. After 6 weeks, although no statistically significant differences were found between the groups for tumor growth or survival, access to a running wheel changed many tumor charactetistics. The active group exhibited increased intratumoral vascularization and blood perfusion, but also an increase of hypoxia-inducible factor 1 (HIF-1). In this study, mice were athymic and therefore lacking T cells, which may explain why tumor growth and overall survival was not affected. Indeed, even more encouraging results have been shown by another study of a very similar design but with immunocompetent animals. Mice allocated to a voluntary exercise condition were compared to a control group (n = 11–12 per group) and it was shown that the exercise group had a significantly lower tumor growth rate (P < 0.012), higher tumor apoptosis (P = 0.048), greater microvessel density (P = 0.004) and increased tumor vessel maturity, as determined by colocalization of CD31 with desmin (281). However, different to the previous study, intratumoral hypoxia was significantly reduced in the active group compared to the control group (P = 0.012). Most importantly, this study examined interaction between exercise and treatment. Tumor bearing mice were allocated to either recieve no treatment, exercise only, cyclophospamide only, or exercise combined with cyclophosphamide (n = 17 per group). It was shown that the combination of exercise and cyclophosphamide had the most striking impact on slowing tumor growth, providing initial evidence that exercise and the adaptations that may follow, improve the delivery of chemotherapy to tumors.

Similar to an improvement in the delivery of drugs to sites where they are needed, physical activity may also enhance the ability of immune cells to migrate to tumors. For example, one study examined a number of cancer models in mice, including breast cancer. Mice were randomized to four weeks of voluntary wheel running, or to a non-running control group prior to tumor cell inoculation. Additional groups were designed to examine questions related to the timing of exercise relative to tumor formation (284). Overall, physical activity resulted in a significant accumulation of tumor infiltrating immune cells, including natural killer cells, CD3+ T cells and dendritic cells, which appeared to be mediated, at least among natural killer cells, by IL-6 and epinephrine. Physical activity was also linked with an upregulation of pathways associated with inflammation in the tumor (e.g., increased gene expression for IL-1-beta, IL-6, TNF-alpha) and immune function (e.g., increased gene expression of NKp46, NKG2D, CD68, CD209, CD8, CD74, FoxP3). Other studies have shown that reduction of hypoxia can also facilitate the infiltration of these immune cells is tumors in mice (285), and given that exercise has been shown to reduce tumor hypoxia, this might be another exercise-induced mechanism that facilitates the homing of immune cells to tumors. However, although some tumor infiltrating lymphocytes may have a beneficial role (e.g., CD8+ T cells) in tumor control (286), other cells, such as myeloid derived suppressor cells could have the opposite effect promoting tumorigenesis, tissue-destruction and metastases (287).

There are likely to be many other characteristics of tumors that could be affected by physical activity or exercise, but the effects on treatment and clinical outcomes may remain unknown. For example, one study has indicated that exercise reduces oxidative stress in breast tumors, as shown by 3-fold lower levels of 8-oxo-dG—a marker of oxidative damage to DNA—when examining tumors from a group of mice that had access to a running wheel compared to controls (288). It has also been hypothesized that physical activity and exercise may counter the dysregulated energy metabolism of cancer cells, which is characterized by high glucose uptake and glycolysis (289). Studies in rats injected intraperitoneally with the carcinogen 1-methyl-1-nitrosourea showed that rats with free access to running wheels exhibited less cancer incidence and a lower average number of tumors per rat compared to controls (290). The exercising rats also showed changes in blood levels of hormones and growth factors involved in glucose metabolism, as reductions in plasma insulin, insulin-like growth factor 1 (IGF-1) and leptin were shown. In support, breast cancer bearing mice undergoing 7 weeks of endurance exercise training studied showed that in addition to a reduction in tumor mass, there was also a significant decrease in the levels of tumor lactate compared to untrained controls (291). Exercise training also resulted in significant changes in the levels of some enzymes that are essential for sustaining a glycolytic phenotype of tumor cells. For example, lactate dehydrogenase isoforms A and B, and monocarboxylate transporter 1 were decreased in tumors from trained mice, which, in combination with lower lactate production, could contribute to slower tumor progression. Indeed, excess of lactate anaerobic metabolism in cancer cells has been associated with poorer activation, infiltration and function of immune cells within the tumor (292). Therefore, these metabolic findings support the positive impact of exercise in enhancing anti-cancer immunity that may improve treatment outcomes.

Despite some very advanced studies with animal models, mechanistic research with human participants examining the effects of exercise on tumor characteristics and clinical outcomes is limited. Indeed, most mechanistic insight in human settings is limited to review articles, which summarize that better clinical outcomes among more active patients, are likely to be linked to mechanisms related to metabolic growth factors, inflammation, immune function, myokines and adipokines (293). Indeed, some understanding of how exercise and physical activity can affect tumors directly comes from studies that have incubated cancer cell lines with human serum collected before and after exercise. For example, a study collected serum from breast cancer survivors before and after a 6-month exercise training intervention (i.e. to examine chronic effects of exercise) and before and after a 2 hour bout of exercise (i.e. to examine acute effects) (294). Breast cancer cell lines were grown in human serum for 48 hours and the effects on viability was examined. Serum samples collected before and after the exercise training intervention provided evidence of a reduction in systemic inflammation shown by lower IL-6 and TNF-alpha post-intervention, but these serum samples had no anti-growth effect on the breast cancer cell lines. However, serum samples collected immediately after an acute bout of exercise—which, as expected, exhibited a high concentration of adrenaline, noradrenaline, lactate and IL-6—reduced the viability of the breast cancer cell lines by approximately 9% (294). Subsequent work showed that breast cancer cells grown in this acute-exercise-conditioned serum were 50% less tumorigenic when implanted into mice, due to adrenaline and noradrenaline activating the Hippo signaling pathway, and subsequent phosphorylation of the YAP protein, reducing the expression of genes associated with proliferation (295).

Prospective cohort studies with patients are ongoing, such as the AMBER study, which is examining relationships between physical activity and health related fitness with treatment outcomes among 1500 newly diagnosed breast cancer patients (296). Physical activity is measured objectively using wearable devices, cardiorespiratory fitness is assessed directly, along with body composition using dual x-ray absorptiometry, and clinical measurements such as lymphedema and fatigue are also being recorded. However, most importantly, molecular measurements in tumors will be interpreted alongside clinical outcomes, with follow up at 1, 3 and 5 years. Among the very few studies which have investigated the relationship between exercise and treatment outcomes with cellular and molecular measurements, is a randomized clinical trial of 20 breast cancer patients undergoing neoadjuvant chemotherapy (297). One group underwent a standard period of doxorubicin and cyclophosphamide treatment, whereas another group received this chemotherapy with supervised aerobic exercise training. Exercise reduced systemic inflammation, but increased some angiogenic factors, including proangiogenic factor placenta growth factor (PLGF). In addition, circulating endothelial progenitor cells increased, which might contribute toward tumor vessel normalization and the reduction of hypoxia, shown by animal studies. However, this study was unable to examine whether exercise improved the clinical response to chemotherapy due to power.

Other human studies provide more indirect evidence of exercise-induced mechanisms that might benefit patients with cancer. For example, it is very well established that acute bouts of exercise cause a transient lymphocytosis and a subsequent lymphocytopenia in the hours after, whereby lymphocytes with strong tissue-migrating and effector capabilities, migrate to peripheral tissues searching for antigens (298). This effect is particularly marked among T cells and natural killer cells, and is thought to represent immunosurveillance, that may even facilitate the detection and elimination of tumors (42, 298–300). The concept that regular exercise might bolster aspects of immune function has been shown by a randomized and controlled trial in breast cancer survivors (301). Participants were randomized to either aerobic exercise training for 15 weeks three times per week (n = 25), or an inactive control group (n = 28). The results showed that regular exercise increased cytotoxic activity of natural killer cells. Other indirect effects of exercise shown in human studies that might benefit patients with cancer might be brought about by interaction with age-related processes, such as immunosenescence and inflammaging. For example, exercise training or remaining physically active throughout life might prevent, limit, delay or even reverse some aspects of immunosenescence (190, 299, 302). A potential mechanism is limiting the expansion of late-stage differentiated T cells by exercise mobilizing these cells to peripheral tissues, where they are exposed to apoptotic signals, followed by a mobilization of hematopoietic cells and trafficking to the thymus, stimulating development of naïve T cells (190). This hypothesis is supported by several observational studies, including a comparison of 125 regular cyclists (55–79 years), 75 age-matched older adults and 55 young adults who did not exercise regularly (303). Cyclists exhibited many features of a less-aged immune system, including lower proportions of late-stage differentiated T cells, high frequencies of B cells, lower levels of IL-6, and higher levels of the thymoprotective cytokine IL-7 (303). In support, another study has shown that higher levels of directly measured cardiorespiratory fitness are associated with lower frequencies of late-stage differentiated T cells and higher frequencies of naïve T cells (304). Finally, it is well established that regular exercise and physical activity can counter inflammation, and perhaps over a lifetime, this effect limits inflammaging (42, 299, 300). For example, a study of 3075 participants aged 70–79 years reported lower levels of inflammatory markers, including IL-6, TNF-alpha and CRP, among those who performed higher levels of exercise (305).

### Adiposity Is Associated With Poor Cancer Outcomes

Overweight and obesity are characterised by excess accumulation of adipose tissue and are commonly been defined using Body Mass Index (BMI), of between 25–30kg/m^2^ or more than 30kg/m^2^ respectively (306). Being overweight or obese is associated with an increased risk of developing breast cancer, and these associations are strongest in postmenopausal women (307, 308). However, a higher BMI and/or higher percentage body fat are measurements that have also been associated with worse clinical outcomes among women diagnosed with breast cancer, including worse prognosis, higher risk of recurrence, and lower overall and disease-specific survival (38–40). For example, a metanalysis showed that there appears to be a linear relationship between BMI and mortality beginning from 20 kg/m^2^ when assessed before diagnosis and up to 12 months after (40). Moreover, obesity also appears to have an impact on the effectiveness of some treatments. A pooled study compared data from 8 prospective trials of breast cancer patients treated with neoadjuvant chemotherapy and found that high BMI negatively influenced the response to anthracycline-taxane based treatment, and was significantly associated with lower rates of pathological complete response (309). High BMI was also associated with shorter disease-free survival and overall survival independently of pathological complete response in luminal-like tumors and in triple negative breast cancer. In addition, obesity has been linked to the development of tumor metastases (310) and recurrence (311). For example, in a study of 1250 HER2 positive breast cancer patients it was shown that in the ER negative subgroup of patients, obese individuals were more likely to develop distant metastases at 5 years (33.4%, 95% CI: 22.1–50.5) than those in the overweight (17.9%, 95% CI: 12.3–25.9) or under/normal weight (17.5%, 95% CI: 13.8–22.4) groups (310). However, not all studies evaluating the influence of overweight and obesity in cancer settings have reported worse outcomes compared to lean counterparts: this phenomenon has been named the “obesity paradox” as some studies reported that people with a high BMI responded better to therapy than expected or had better survival rates (312). As an example, a prospective study of 88 metastatic breast cancer patients on palliative chemotherapy analyzed the impact of BMI on survival and treatment response over a follow up period of 40 months (313). It was shown that a greater proportion of overweight patients were most responsive to treatment (56%) followed by obese patients (30%) compared to a smaller proportion in the normal weight group (15%) (313). Moreover, patients with a BMI ≥25 kg/m^2^ survived for longer (19 months) in comparison with patients who had a BMI < 25 kg/m^2^. However, it is worth considering that this study has a relatively small sample size and it may not have adequately controlled for other potentially influencing factors, such as tumor type, receptor status, extent of disease, cardiovascular risk, etc.

### Adiposity Could Be Associated With Poor Cancer Outcomes Due to Undertreatment

It has been suggested that in the past, obesity has been linked with undertreatment, where the dose of some chemotherapies has been adjusted to the ideal body mass of a patient, or arbitrarily capped at a body surface area of 2.0 m^2^. For example, a retrospective cohort study compared treatment patterns among overweight, obese, and patients of a normal weight, in a total of 9672 breast cancer patients treated with chemotherapy (314). The results showed that, compared to the 9% of people in the healthy weight group, 11% of the overweight group, 20% of the obese group, and 37% of the severely obese group, were administered dose reductions during their first chemotherapy cycle. This reduction in the dose has been associated with poorer outcomes (315), and could partially explain why adiposity relates to worse prognosis. The rationale for dosing chemotherapy based on body surface area, rather than absolute body mass, is to avoid toxicity, however evidence shows that this strategy could lead to poor clinical outcomes and that toxicity is unlikely. For example, a study examined data from 1,435 stage II breast cancer patients undergoing adjuvant chemotherapy to determine if dosing based on actual body mass increased risk of toxicity (316). Analyses during the first chemotherapy cycle showed that patients with a BMI ≥ 27.3 kg/m^2^ who were dosed according to actual body mass did not exhibit excess toxicity (% of women with toxicity: 47% of overweight women vs. 51% of lean women, P = 0.51). Indeed, compared to overweight women who received a dose reduction due to body surface area dosing, overweight women who received their dose based on actual body mass, had an adjusted risk ratio of treatment failure of 0.73 95% CI: 0.53–1.00, indicating that dose reduction can lead to poor clinical outcomes. However, guidelines now advocate dosing chemotherapy for obese patients based on absolute body mass (317, 318). Thus, understanding why obesity is associated with poor treatment outcomes, requires further investigation.

### Adiposity Influences Cancer Biomarker Profiles

Obesity is associated with particular molecular subtypes of breast cancer. For example, a study evaluated the link between BMI and breast cancer subtypes (319). In a retrospective analysis of 848 patients with primary operable breast cancer, groups were formed on the basis of BMI: normal weight (BMI = 18–24.9 kg/m^2^), overweight (BMI = 25–29.9 kg/m^2^) and obese (BMI > 30 kg/m^2^). The results showed that triple negative breast cancer was more common among overweight and obese women, whereas HER2-positive tumors were more frequent among women of normal weight.

Body composition can also affect the properties of tumors, as well as the levels and characteristics of some cancer biomarkers. Evidence in support comes from randomized and controlled trials implementing behavioral or lifestyle interventions to bring about changes to physiology. For example, one study randomized 32 overweight or obese stage 0-II breast cancer patients into an intervention and control group as part of a 30 day pre-surgery “weight loss” study (320). The intervention group received counseling on caloric restriction and aerobic exercise to promote a change in body mass of 0.68–0.92 kg/week. The control group received nutritional counseling and upper body resistance exercise which was assumed to elicit a smaller energy expenditure than aerobic exercise. Circulating cytokines and metabolic measurements implicated in cancer progression but also tumor characteristics were assessed. The intervention group exhibited a greater change in body mass than the control group (−3.62 vs. −0.52 kg) and exhibited greater changes in metabolic measurements, including serum leptin and fasting insulin, and inflammatory markers such as TNF-alpha. Most importantly, a greater change to body mass and accelerometer-measured physical activity was positively associated with an infiltration of the CD56+dim cytotoxic sub-population of natural killer cells into tumors. Indeed, tumors from the intervention group were characterized by a greater expression of key genes associated with immune cell recruitment (e.g., CX3CL1, CXCL1, and CXCL12), and higher TNF-alpha, but there were no differences in Ki-67 between groups.

Other evidence for body composition affecting cancer biomarkers comes from cross-sectional studies. For example, one study investigated the association between BMI in 535 post-menopausal women with operable breast cancer and the expression of HER2. The results showed that, with increasing BMI, there was a significant decrease in HER2 overexpression (321). The circulating form of HER2 has also been shown to be positively associated with BMI in a healthy population of males and females aged 45–65 years (322). Other cross-sectional studies have examined the influence of BMI on results from molecular profiling tests. For example, 865 postmenopausal women with breast cancer were divided into groups on the basis of BMI (<25 kg/m^2^, 25–30 kg/m^2^ or ≥30 kg/m^2^). It was shown that IHC4 and Oncotype RS had the most prognostic value for distant recurrences in the group with the lowest BMI and there was no prognostic value in the group with a BMI ≥30 kg/m^2^. In the case of Prosigna, the score was most prognostic in patients with a BMI 25–30 kg/m^2^. Other cross-sectional studies have examined TILs in the context of body composition. For example, functional tumor infiltrating CD8+TILs were assessed in two groups of breast cancer patients who were classified as either lean (BMI < 25 kg/m^2^) or obese (BMI > 32.5 kg/m^2^). It was shown that CD8+ TILs from obese patients had a significantly lower expression of Granzyme B (323). Furthermore, there was a significantly lower number of these cells in the lymph nodes draining the tumor in the obese group.

Other studies have examined soluble cancer biomarkers in a variety of body fluids. For example, a study of 128 women with breast cancer (89 post-menopausal) and 254 without breast cancer (125 post-menopausal) measured prostate specific antigen (PSA) in serum and nipple aspirate fluid (324). Among women with breast cancer, PSA measured in nipple aspirates from pre-menopausal women negatively correlated with BMI (r = −0.53, P = 0.049), whereas PSA correlated positively with BMI in samples from post-menopausal women (r = 0.37, P = 0.017). Among women without breast cancer, serum PSA was negatively correlated with BMI in both pre- (r = −0.56, P = 0.001) and post-menopausal women (r = −0.37, P = 0.017), but this association was lost when controlling for plasma volume (324). Indeed, obesity is associated with an expansion of blood and plasma volume (325–327) and it is often not considered that the concentration of cancer biomarkers reported in cross-sectional studies could be affected. For example, a study investigated the effect of plasma hemodilution on the concentration of several tumor markers in 6917 healthy women and found that BMI was significantly positively associated with a greater plasma volume, as well as with higher serum concentrations of CEA and α‐fetoprotein and lower concentrations of CA 125 and CA 19.9 (328). Even in investigations examining changes over time with serial measurements, results might be affected by shifts in plasma volume. Bouts of exercise that could have been undertaken by study participants and patients in the hours before blood sampling, which is sometimes not controlled for, can decrease plasma volume by up to about −10%, artificially increasing the concentration of some measurements (329, 330). Although these potential inaccuracies in reported values are probably only a minor consideration, they could shift a measurement above or below a cut-off or threshold that influences treatment decisions, or with serial measurements, could give falsely influence estimates of disease progression.

### Adiposity Can Affect Tumors Directly and Indirectly

The mechanisms underlying links between obesity and breast cancer treatment have not been determined. Some mechanisms could be indirect and systemic due to the impact that overweight and obesity has on metabolic health, inflammation, and immune competency, whereas other mechanisms could be more direct, or at least related to the characteristics of local tissue surrounding breast tumors. Adipose tissue could in principle contribute to local tumorigenesis, but perhaps counter-intuitively, women with a high percentage of breast adipose tissue, are at a lower risk of disease (331). Indeed, high mammographic density, characterized by radiologically dense breasts consisting of epithelial or stromal tissue which appears light on a mammogram, compared to adipose tissue which appears dark, is a strong predictor of breast cancer risk (332, 333). Although BMI and physical activity should be considered when interpreting mammographic density data (334, 335) it is important to emphasise that the characteristics of breast adipose tissue, such as the phenotype, and the secretory profile, are probably the most important factors that could influence breast tumors.

*In vitro* and *in vivo* animal studies have examined whether interactions between breast cancer cells and different cell types within surrounding adipose tissue, such as mature and immature adipocytes, and normal and cancer associated fibroblasts, influence tumor progression (336, 337). Using cell co-cultures and mouse models, it was shown that cancer cells triggered phenotypical changes in the surrounding adipocytes, such as increased production of proteases and pro-inflammatory mediators including IL-6, IL-8, CCL2 and CCL5 (336). Indeed, this cross-talk between so-called cancer-associated adipocytes, contributed toward cancer progression and invasion (336). Cytokine production was enhanced further when cancer cells interacted with immature adipocytes stimulating mammosphere formation, resulting in higher invasion and metastatic potential. Indeed, when the cancer cells were injected into mice after co-culture with immature adipocytes for 7 days, the number of tumor initiating cells increased 3-fold, and the volume of metastases in the lungs increased as did the number of circulating tumor cells (337). Further experiments showed that immature adipocytes and the release of cytokines upregulated embryonic stem cell transcription factors c-MYC, SOX2, and NANOG, through Src activation, promoting the expansion of cancer stem cells (337).

Other animal studies have shown that adipocytes from human and mouse breast tissue recruit and activate macrophages (338). For example, one study has used a human-in-mouse breast cancer model whereby human breast adipose stromal cells, modified to model an inflammatory environment of obese breast, are injected into the mouse mammary fat. In this work, mice were randomized to eat either a normal diet (ND) or to eat a diet with increased calories from fat (HFD). It was shown that in mammary glands of HFD mice, total numbers of macrophages were significantly increased (4.4 x10^5^ ± 0.5 x 10^5^; macrophages/gland) compared with ND mice (2.5 x10^5^ tumor± 0.5 x10^5^; P = 0.05). It was also shown that the recruitment and activation of these macrophages was through the CCL2/IL-1b/CXCL12 signaling pathway. These findings provide a mechanistic role for adipocytes leading to adipose tissue dysfunction in breast tissue, which could precede tumor development (338). A study in mice evaluating obesity-promoted breast tumor growth showed that increased oxidation of fatty acids and reduced glycolysis, both enhanced by the leptin-PD-1-STAT3 axis in CD8+ TILs, promoted obesity-related breast tumorigenesis and contributed to resistance to immunotherapy (323). Inhibiting STAT3 or fatty acid oxidation restored CD8+ T cell effector functions and inhibited tumor development in obese mice. Other murine studies have provided further evidence that obesity can impair cancer immune surveillance. For example, showing that obesity promotes hyperactivation of CD8+ TILs, and an accumulation of granulocytic myeloid-derived suppressor cells (G-MDCSs), which induced Fas/FasL mediated apoptosis of CD8+ T (339).

Research in humans has also examined links between breast cancer and dysfunctional adipose tissue. For example, one study compared two groups of individuals without a breast cancer diagnosis (lean n = 37, obese n = 19) to patients with breast cancer (n = 12) (340). Using RT-PCR to examine expression levels of genes in circulating leukocytes, it was shown that TNF-alpha, IL-6, leptin and ErbB2, were significantly higher in obese individuals without a cancer diagnosis and among breast cancer patients compared to the lean group. Assuming leukocyte gene expression of ErbB2 is representative of gene expression in breast tissue, then obesity-associated over-expression could have important implications for tumorigenesis and treatment, given its role in metastatic disease. A possible mechanism underlying interactions between disease progression and adipose tissue surrounding breast tumors could be the adoption of an adipose derived secretory phenotype that attracts different populations of immune cells. Adipose tissue dysfunction is characterised by changes to the tissue microenvironment at cellular and structural levels, which results in abnormal secretions derived from adipocytes and local immune cells (197). Changes include adipocyte hypertrophy, hypoperfusion, hypoxia and impaired insulin signaling, leading to an enlargement of adipose tissue, low-grade systemic inflammation due to the release of inflammatory cytokines (341, 342) and possibly exacerbated immunosenescence (343). These changes lead to immune cell accumulation within adipose tissue, most prominently consisting of macrophages with a pro-inflammatory phenotype and effector-memory CD8+ T cells (195, 197). The implications of attracting highly inflammatory populations of immune cells to areas surrounding breast tumors are unknown, but could conceivably have both negative and positive effects, depending on the cell type recruited, perhaps in part providing one explanation for the “obesity paradox”. For example, a study investigated 334 breast tumors from patients with long-term follow-up and showed that high frequencies of tumor infiltrating CD8+ T cells were associated with higher cumulative breast cancer specific survival (344). On the other hand, a metanalysis of sixteen studies and a total of 4,541 breast cancer patients showed that overall survival and disease free survival correlated with high frequencies of tumor associated macrophages (overall survival: HR = 1.50, 95% CI: 1.20–1.88 vs. disease free survival: HR 2.23, 95% CI: 1.72–2.90) (345).

Although in obesity, there is often a large accumulation of abdominal adipose tissue, deposition occurs elsewhere, including the breast, and a question that remains is whether regional depots of adipose tissue interact differently with tumors. To further improve our understanding of this question, a study isolated breast tissue-derived and abdominal tissue-derived mesenchymal stem cells (MSCs) from healthy adults undergoing cosmetic surgery (346). MSCs, with the capacity to differentiate into adipocytes, were co-cultured with MCF7 or MDA-MB-231 breast cancer cell lines and compared to co-culture with human macrophages. MSCs from both regions stimulated proliferation of the breast cancer cell lines similarly, and abdominal MSCs had a higher expression of IL-1-beta compared to breast MSCs. Co-culturing MSCs with macrophages led to higher levels of VEGF-A, VEGF-C, SER-PINE1, FGF2, IL-1-beta and IL-6 gene expression in macrophages. Thus, MSCs, and perhaps adipocytes from both breast and abdominal depots, interact with macrophages, which could lead to the development of dysfunctional adipose tissue.

In summary, further studies are required to understand mechanistic interactions between adipose tissue—including adipocytes and adipose-associated immune cells—with breast cancer cells. Indeed, if the dysfunction of adipose tissue surrounding breast tumors influences the accumulation of local immune cells, tumor infiltrating lymphocytes, and other tumor characteristics, then this process could have an impact on the expression of tumor biomarkers and cancer progression. Moreover, systemic adipose tissue dysfunction could lead to metabolic, inflammatory and immunological profiles that have been associated with poor clinical outcomes. Encouragingly, if adipose tissue dysfunction and adipose derived secretions contribute to tumorigenesis, then lifestyle interventions could in principle limit disease progression and facilitate treatment. For example, regular exercise, triggers a reduction in fat mass and limits the release of adipokines, resulting in anti-inflammatory adaptations (42, 299, 347).

## Conclusions

**Figure 1:**
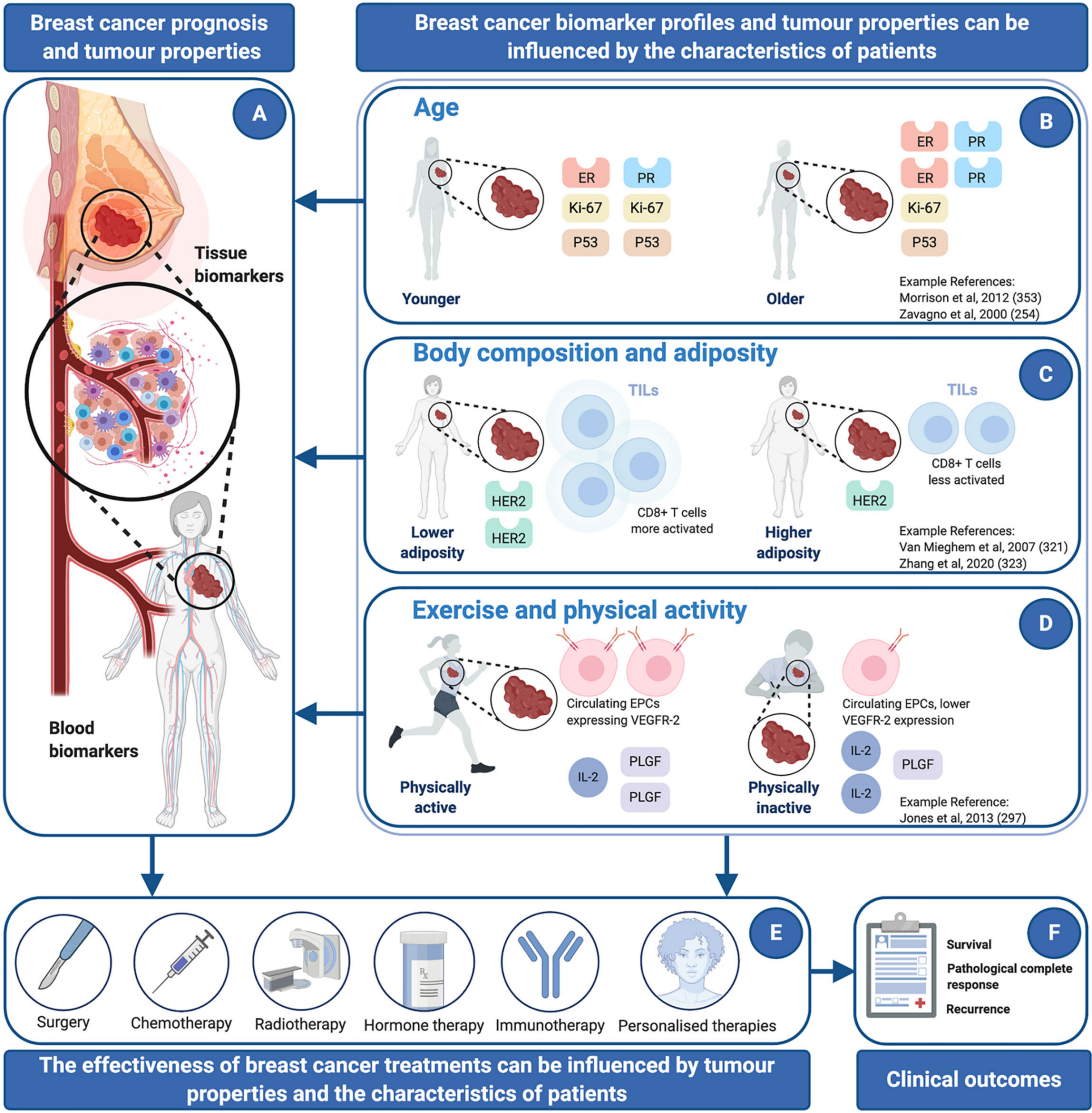
Breast cancer prognosis, tumor properties, and clinical outcomes can be influenced by the characteristics of patients, including: age, body composition and adiposity, or exercise and physical activity. References are considered to be representative examples of robust human studies with breast cancer patients. **(A)** Cancer biomarkers can be assessed in tumor tissue or in blood and can provide information about prognosis and the clinical response to different treatments. **(B)** Some studies have shown that older age is associated with lower expression of tumor proliferative markers (e.g., Ki-67) and proteins implicated in tumor progression (e.g., P53), and higher expression of certain hormone receptors (e.g., ER, PR). **(C)** Higher adiposity has been associated with a lower expression of HER2, a lower magnitude of tumor immune cell infiltration and lower activation status of tumor-resident CD8+ T cells. **(D)** Bouts of exercise and physical activity have been shown to decrease some inflammatory markers (e.g., IL-2) and increase pro-angiogenic factors (e.g., PLGF and EPCs expressing VEGFR-2). Higher tumor vascularity could facilitate the delivery of drugs to a tumor. **(E)** The effectiveness of breast cancer treatments can be influenced by tumor properties [shown in panel **A**] and the characteristics of patients [shown in **(B–D)**]. **(F)** In turn, interaction between tumor properties, the characteristics of patients, and the effectiveness of breast cancer treatments can influence clinical outcomes. EPCs: Epithelial Progenitor cells, ER: Estrogen Receptor, HER2: Human Epidermal Growth Factor Receptor-2, IL-2: Interleukin 2, IL-6: Interleukin 6, KI-67: nuclear protein Ki-67, PGLF: Placenta Growth Factor, PR: Progesterone Receptor, P53: tumor protein 53, TILs: tumor Infiltrating Lymphocytes, VEGFR-2: Vascular Endothelial Growth Factor Receptor-2. Figure created with BioRender.com. Adapted from “tumor Microenvironment 2” and “Types of Cancer Treatment”, by BioRender.com (2020). Retrieved from https://app.biorender.com/biorender-templates.

Managing heterogeneity in the clinical response exhibited by patients remains a challenge. The first part of this article summarized biomarkers that are available to address this problem, by informing therapeutic options, assessing pathological response and predicting clinical outcomes. The second part of this article summarized factors such as aging, physical activity, and body composition, that might influence the sensitivity and specificity of these biomarkers, by modulating the cellular composition and function of tissues. This article has highlighted that the characteristics of patients, including their age, physical activity level and adiposity, could interact with disease progression and influence treatment effectiveness due to a combination of direct and indirect mechanisms (**Figure 1**). Indeed, processes and profiles associated with lifestyle, including metabolic health, inflammaging and immunosenescence, are gaining increasing recognition as being important factors that can influence cancer and its treatment. The positive outlook is that some of these processes might be reversible, or at least, their development might be slowed or limited, by for example, encouraging patients to lead a physically active lifestyle, at almost any stage of disease. In summary, the measurement of cancer biomarkers in blood or in tumors could be influenced by patient characteristics and their lifestyle, because these factors affect the composition and function of cells and tissues across the body and across the life-course. These factors are not commonly considered clinically or in research, either for practical reasons or because the supporting evidence base is developing. Thus, a broader perspective within cancer care is required which integrates objective measurements of aging, lifestyle and other patient characteristics, using a combination of established biomarkers measured in tissues and in blood, but also broader whole-body measurements of physical functioning and frailty (216, 219, 220). Given the literature presented herein, we hope that this article encourages an interdisciplinary phenomic approach in oncology research and clinical management.

## Author Contributions

JT and AAE conceived the idea, drafted the manuscript and critically appraised evidence. MB, JC, RJ, RB, KG, PB, and DT undertook a critical review of the manuscript, edited, and contributed toward writing. All authors contributed to the article and approved the submitted version.

## Funding

This work was supported in part by grant MR/N0137941/1 for the GW4 BIOMED DTP, awarded to the Universities of Bath, Bristol, Cardiff and Exeter from the Medical Research Council (MRC)/UKRI.

## Conflict of Interest

The authors declare that the research was conducted in the absence of any commercial or financial relationships that could be construed as a potential conflict of interest.

## Footnotes

1 It should be considered that unless the relationship between a biomarker and disease is causal, then a change to biomarker concentration might not necessarily reflect a prognostic (or predictive) change.
